# New species of *Bidessonotus* Régimbart, 1895 with a review of the South American species (Coleoptera, Adephaga, Dytiscidae, Hydroporinae, Bidessini)

**DOI:** 10.3897/zookeys.622.9155

**Published:** 2016-10-06

**Authors:** Kelly B. Miller

**Affiliations:** 1 Department of Biology and Museum of Southwestern Biology, University of New Mexico, Albuquerque, NM 87131-0001 USA

**Keywords:** Water beetles, taxonomy, classification, Neotropical, Bidessonotus, Dytiscidae, Coleoptera

## Abstract

The South American species of the New World genus *Bidessonotus* Régimbart, 1895 are reviewed with descriptions of seven new species. This brings the total number of valid *Bidessonotus* species to 37, making it the largest Bidessini genus in the New World. The new species are *Bidessonotus annae* **sp. n.** (Venezuela), *Bidessonotus josiahi* **sp. n.** (Venezuela), *Bidessonotus palecephalus* **sp. n.** (Venezuela), *Bidessonotus reductus* **sp. n.** (Venezuela), *Bidessonotus septimus* **sp. n.** (Venezuela), *Bidessonotus spinosus* **sp. n.** (Venezuela), and *Bidessonotus valdezi* **sp. n.** (Guyana, Suriname). New distribution records are provided for many other South American *Bidessonotus* species. The main diagnostic features of *Bidessonotus* species are in the male genitalia, and these are illustrated for all South American species. Diagnostic features, distributions (including distribution maps), and additional comments are provided for all South American species.

## Abstract (es)

Las especies sudamericanas del género del Nuevo Mundo *Bidessonotus* Regimbart, 1895 (Coleoptera: Adephaga: Dytiscidae: Hydroporinae: Bidessini) se revisan con descripciones de siete especies nuevas. Se eleva el número total de especies válidas en *Bidessonotus* a 37, el género más grande de Bidessini en el Nuevo Mundo. Las especies nuevas son *Bidessonotus annae* **sp. n.** (Venezuela), *Bidessonotus josiahi* **sp. n.** (Venezuela), *Bidessonotus palecephalus* **sp. n.** (Venezuela), *Bidessonotus reductus* **sp. n.** (Venezuela), *Bidessonotus septimus* **sp. n.** (Venezuela), *Bidessonotus spinosus* **sp. n.** (Venezuela), y *Bidessonotus valdezi* **sp. n.** (Guyana, Surinam). Se incluyen nuevos registros distribucionales para la mayoría de las otras especies de *Bidessonotus* en América del Sur. Los principales caracteres diagnósticos para las especies de *Bidessonotus* están en los órganos genitales de los machos, los cuales se ilustran para todas las especies sudamericanas. También se incluyen caracteres diagnósticos, mapas de distribución, y comentarios adicionales para todas las especies sudamericanas.

## Introduction

The New World Bidessini genus *Bidessonotus* Régimbart (Coleoptera: Dytiscidae: Hydroporinae) included, prior to this paper, 30 species, making it one of the largest genera in the tribe in the New World with only *Neobidessus* Young (30 species) and *Liodessus* Guignot (27 species) comparable in size. There are three species in eastern and southern North America, numerous species in Central America and the Caribbean and additional species in South America. Although many *Bidessonotus* species are widespread, others are relatively narrowly distributed, and new species are likely to be discovered with additional collecting efforts in undercollected areas of South America.

**Figures 1–4.**
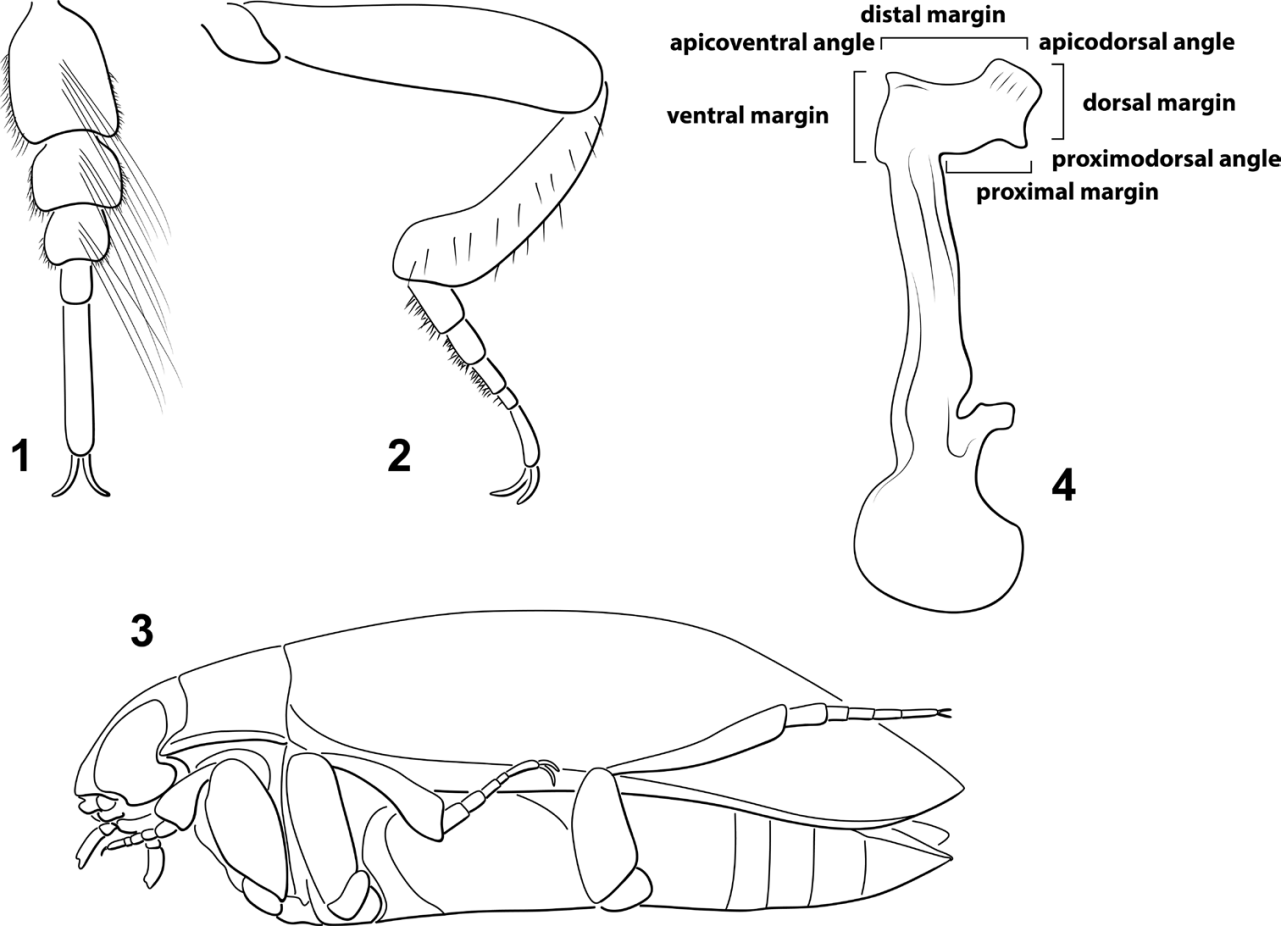
**1–2** *Bidessonotus obtusatus* male. **1** Left protarsomeres, dorsal aspect **2** Left mesoleg, anterior aspect **3** *Bidessonotus tibialis* male, habitus, lateral aspect **4** *Bidessonotus rubellus*, male median lobe, right lateral aspect showing regions referenced in diagnoses and descriptions.

*Bidessonotus* are easily diagnosed by the presence of relatively distinctively pentamerous pro- and mesotarsi (Fig. 1) whereas in other Bidessini these are pseudotetramerous with tarsomere IV small and concealed in the lobes of III. Males are characteristic, too, with strongly curved mesotibiae (Fig. 2) and a distinctly concave ventral surface (Fig. 3). The male median lobe is strongly asymmetrical and laterally flattened with a broadly expanded and characteristically shaped apex (like a “hatchet,” e.g Fig. 4). Species differ especially in the shape of this apex along with body size and coloration. Otherwise, species are relatively similar to each other, typically elongate oval, mottled gray and brown with the head and pronotum yellow, and females of many species, and occasionally males also, purplish iridescent.

The genus was originally described by Régimbart (1895) to include seven species, three of which were new, with a few names treated as junior synonyms. Earlier species were placed in *Hydroporus*, but later in *Bidessus* by Sharp (1882). Blatchley (1919) next described a new species from Florida placing it in *Bidessus*, however J. Balfour-Browne (1947) later transferred it to *Bidessonotus*, who revised the genus clarifying a number of the described species and adding eleven new ones. After this, little was done in the genus for several decades except for one new species described by Guignot (1957). A significant contribution was made, however, by Young (1990), who revised the entire genus and added nine new species. Finally, a new species was described by Miller (1997).

Collecting in poorly known areas of northern South America during the past decade has led to discovery of seven previously undescribed species of *Bidessonotus*, and these are described here. This brings the number of known species to 37. New records of other South American species are also presented here along with brief diagnostic descriptions, illustrations, additional comments, and distributions. Young (1990) was unable to write a suitable key because the diagnostic features of species are coloration, size and male genitalia, and come in such combinations as to make a key difficult. That problem persists, and no key is presented here. The best way to identify species is to compare male genitalia against the illustrations and use distribution information.

## Materials and methods

**Measurements.** Measurements were made with an ocular scale on a Zeiss Discovery V8 dissecting microscope. Emphasis was placed on getting the diagnostic range of measurements of structures rather than finding the average or taking a random sample. Measurements include: 1) total length (TL), 2) greatest width across elytra (GW), 3) greatest width of pronotum (PW), 4) greatest width of head (HW), and 5) distance between eyes (EW). The ratios TL/GW, HW/EW and FW/FL were also calculated.

**Images.** Illustrations were made using a drawing tube on a Zeiss Discovery V8 dissecting scope. Sketches were first done in pencil then scanned, placed into an Adobe Illustrator artboard and “inked” digitally using vector lines. The illustration of the male median lobe of *Bidessonotus bicolor* was redrawn from Guignot (1957).

**Material.** Specimens were examined from the following collections:

CSBD
 Center for Biological Diversity, University of Guyana (type specimens currently reposed with KUNHM) 


FSCA
 Florida State Collection of Arthropods, University of Florida, Gainesville, FL, USA (P. Skelley) 


KBMC
 Kelly B. Miller Collection, Museum of Southwestern Biology, University of New Mexico, Albuquerque, NM, USA 


KUNHM
University of Kansas Natural History Museum, University of Kansas, Lawrence, Kansas, USA (A.E.Z. Short) 


MIZA
 Museo del Instituto de Zoología Agrícola Francisco Fernández Yépez, Universidad Central de Venezuela, Maracay, Venezuela (L. Joly) 


MSBA
Museum of Southwestern Biology Division of Arthropods, University of New Mexico, Albuquerque, NM, USA (K.B. Miller) 


NZCS
 National Zoological Collection of Suriname, Paramaribo, Suriname (P. Ouboter) 


USNM
 United States National Collection of Insects, Smithsonian Institution, Washington, DC, USA (T. Erwin) 


Label data for holotype specimens are reported verbatim. All other label data, including for paratypes, are reported in a standardized format. All paratypes have a blue label with a black line border bearing the species name attached to them.

**Distribution maps.** Dot maps presented here are derived from examined specimens and specific localities reported by Young (1990) and J. Balfour-Browne (1947).

### Taxonomic characters

*Bidessonotus* are superficially extremely similar to each other with a similar body shape and coloration. There are some distinctive character systems, however, that require some explanation.

*Head*. The anterior surface of the clypeus of some *Bidessonotus* has a moderately distinctive transverse shallow sulcus which may be interrupted medially. Young (1990) used this character, but I have found it to be somewhat variable within species and difficult to assess, so it is not emphasized here.

*Pronotum*. The basal pronotal plicae are somewhat variably impressed with some species deeply and others more shallowly. However, Young (1990) found the relative length of the pronotal plicae to the elytral plicae to be about the same length and not variable between South American species, so this comparative feature is not used here.

*Prosternal process*. The prosternal process in *Bidessonotus* is elongate and slender. There is some variability in the relative width and the apex that ranges from sharply pointed to rounded. The surface may be convex to flat to somewhat sulcate, features emphasized by Young (1990), but emphasized less here given lack of variability in the South American species.

*Male genitalia*. Dytiscid male genitalia are rotated in such a way as to make describing orientation of structures difficult. This paper follows Miller and Nilsson (2003) in the way these features are described. The male median lobe is bilaterally asymmetrical with the base typically very large and complex and the apical portion developed into a flattened, dorsally directed, roughly rectangular “blade” (something like a hatchet blade) with a highly variable and species-specific shape that is the most reliable diagnostic feature for *Bidessonotus*. The dorsal, distal, ventral and proximal margins have variable shapes, spines and teeth (e.g. Fig. 4). The lateral lobes are bilaterally asymmetrical with the apical segments, in particular, often species specific and useful for species diagnostics.

*Sexual dimorphism*. All *Bidessonotus* species are distinctively sexually dimorphic. The metaventrite and medial portions of the metacoxae are distinctly concave in males (Fig. 3), possibly to correspond to the convex dorsal surface of females when mating. Also, the male mesotibiae are abruptly curved in males (Fig. 2), but straight in females. Other sexual dimorphisms are more variable. Young (1990) emphasized lateral impressions on abdominal ventrite VI that are more distinctly impressed in males. However, South American species have only indistinct, and more medial, modifications to the ventrite. Females of some species have the elytra more abruptly rounded apically with the apicolateral margins broadly lobed (Figs 6b, 7b) and others have a distinct subapical spine on the margin of the elytron (Fig. 10b) whereas males have the apicolateral margins evenly curved (e.g. Figs 6a, 7a, 10a). Finally, coloration and dorsal microsculpture can be somewhat variable with females often matte and/or dorsally purplish iridescent, though in some cases males may also have such coloration.

**Figures 5–11.**
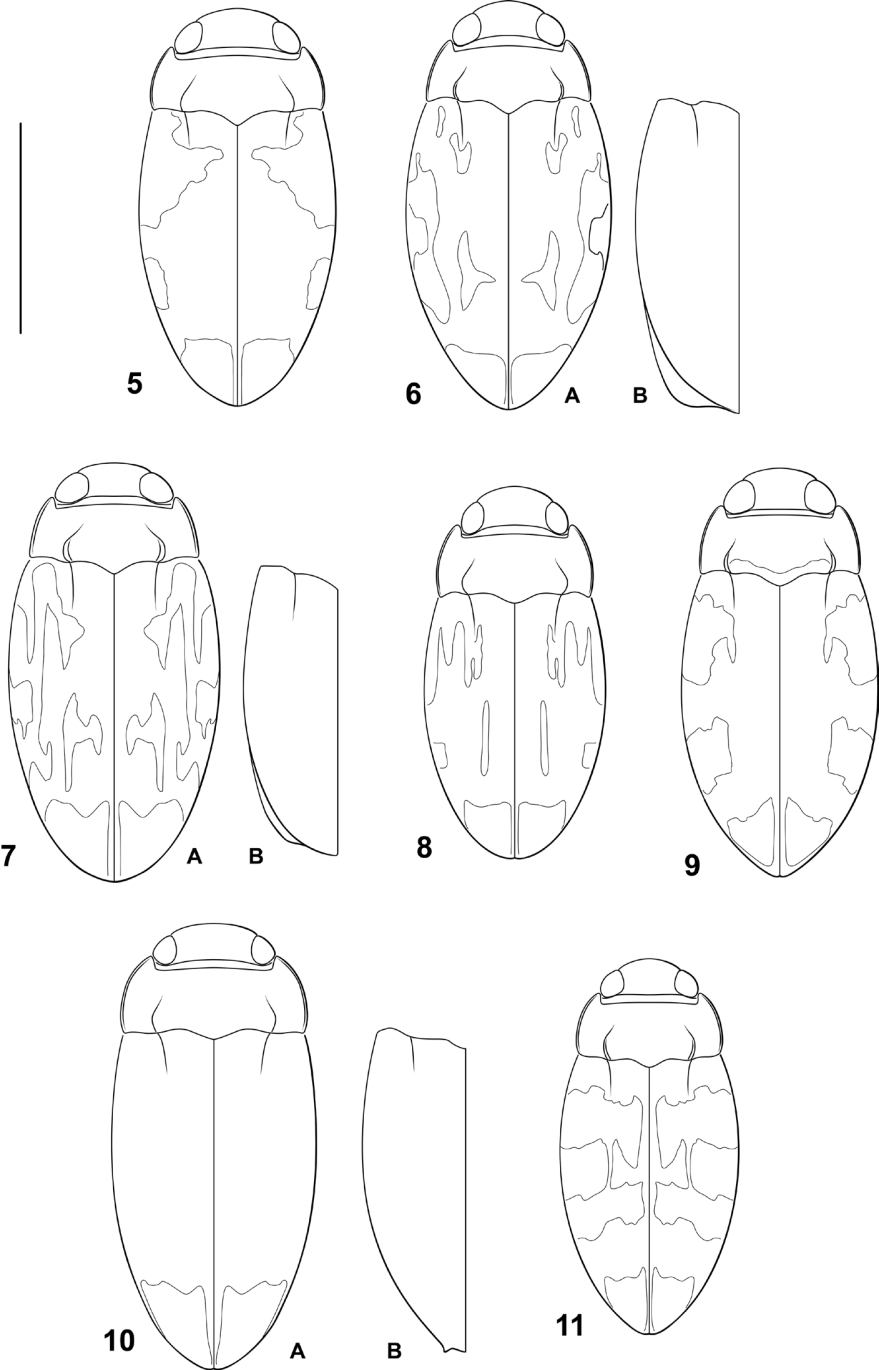
*Bidessonotus* species, dorsal habitus. **5** *Bidessonotus annae* **6** *Bidessonotus josiahi* **A** male **B** female, left elytron **7** *Bidessonotus palecephalus* **A** male **B** female, left elytron **8** *Bidessonotus reductus* **9** *Bidessonotus septimus* **10** *Bidessonotus spinosus* **A** male **B** female, left elytron **11** *Bidessonotus valdezi*. Scale bar: 1.0 mm.

## Taxonomy

### Bidessonotus annae

Taxon classificationAnimaliaColeopteraDytiscidae

Miller sp. n.

http://zoobank.org/9BEB8E25-1EE8-46F8-A78E-F7D7CC5A2ABD

Figs 5 , 12 , 30

#### Type locality.

Venezuela, Apure State, Communidad Caño Gato, on Rio Sipapo, 4°58.838'N, 67°44.341'W.

#### Diagnosis.

**Figures 12–18.**
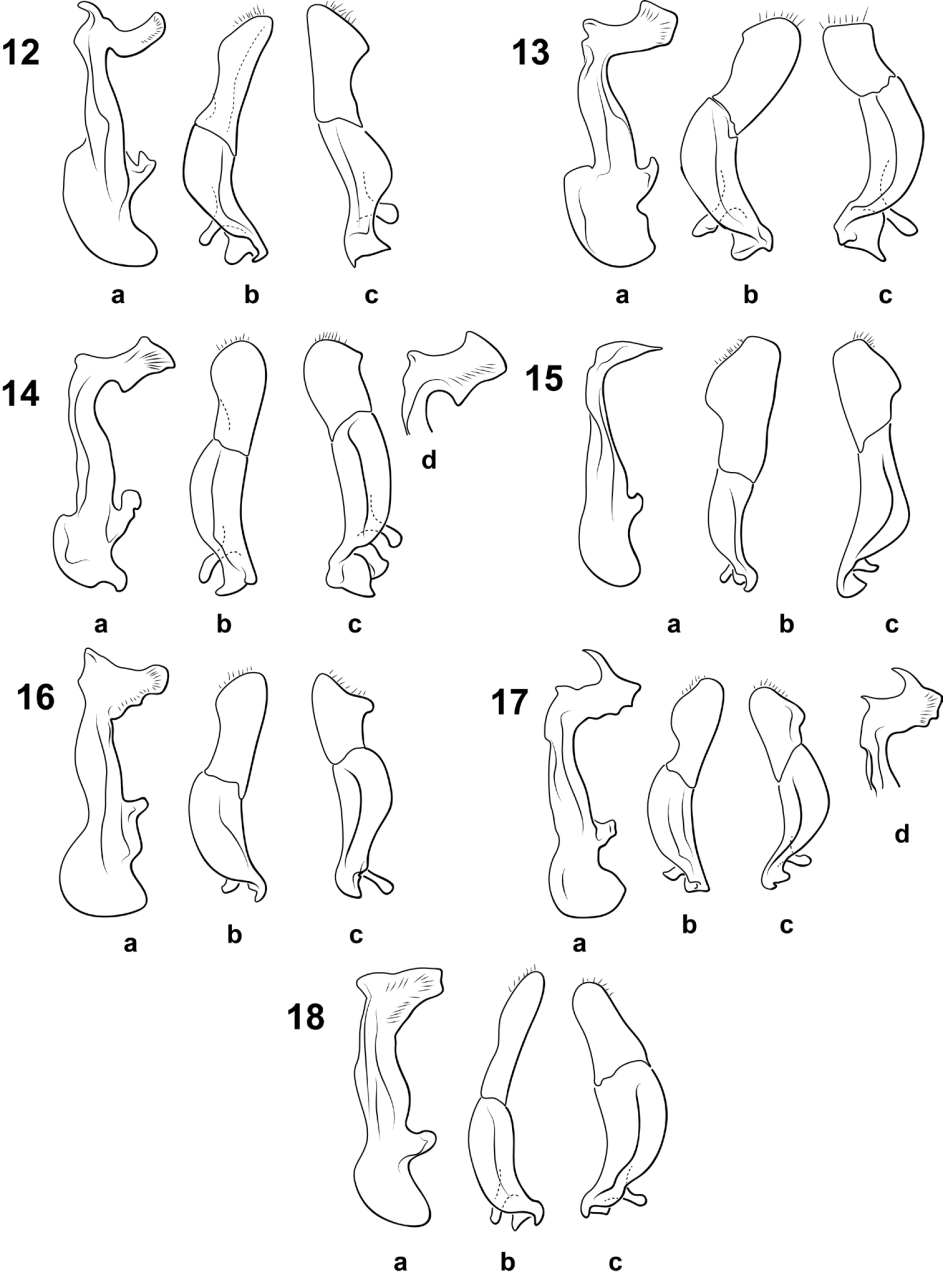
*Bidessonotus* species, male genitalia **A** median lobe, right lateral aspect **B** right lateral lobe, right lateral aspect **C** left lateral lobe, apical segment, left lateral aspect **D** median lobe apex, oblique right lateral aspect **12** *Bidessonotus annae* **13** *Bidessonotus josiahi* **14** *Bidessonotus palecephalus* **15** *Bidessonotus reductus* **16** *Bidessonotus septimus* **17** *Bidessonotus spinosus* **18** *Bidessonotus valdezi*.

Specimens of this species are brown with irregular, indistinct paler regions. The prosternal process is laceolate, shallowly sulcate and apically pointed. The female elytron is unmodified. The apical blade of the male median lobe is slender and curved with an elongate, curved, apically narrowly rounded process at the apicoventral angle, and the dorsal margin narrowly truncate (Fig. 12a). The lateral lobes are nearly bilaterally symmetrical (Fig. 12b, c), though the left lateral lobe has the apex somewhat more broad with a more distinctive, angulate expansion on the ventral margin (Fig. 12c). Male genitalia are similar to those of *Bidessonotus tibialis* but the apical blade in *Bidessonotus annae* is more slender, more strongly curved, and more truncate along the dorsal margin, and the anteroventral process is very slender, strongly curved and directed ventrad whereas it is somewhat broader and directed apically in *Bidessonotus tibialis*.

#### Description.

*Measurements*. TL = 1.7–1.8 mm, GW = 0.8–0.9 mm, PW = 0.7–0.8 mm, HW = 0.5–0.6 mm, EW = 0.3 mm, TL/GW = 2.0–2.1, HW/EW = 1.9. Body shape elongate, lateral outline discontinuous between pronotum and elytron.

*Coloration* (Fig. 5). Head yellow orange. Pronotum yellow orange, darker along posterior margin. Elytron base color orange brown with pale diffuse areas anteriorly and medially, and a distinctive pale macula apically (Fig. 5); without purplish iridescence. Ventral surfaces orange on all surfaces, some sutures darker.

*Sculpture and structure*. Head with anterior clypeal margin evenly rounded; surface smooth and shiny, impuncate, slightly microreticulate; antennomeres III-X moderately broad, slightly asymmetrical. Pronotum widest near posterior angles, lateral margins evenly curved; basal striae moderately impressed, extending anteriorly more than halfway across surface; posterior margins distinctly undulate; surface overall shiny, slightly microreticulate, finely punctate. Elytron with lateral margins broadly curved; basal stria distinct, elongate, moderately impressed; surface of elytron covered with fine punctation, surface between punctures matte, microreticulate. Prosternal process elongate, lanceolate, apically pointed, surface broadly convex throughout length. Metaventrite with distinctive carinae extending from medial apex of metaventrite process posteriorly to near posterior margin at anterior terminus of metacoxal lines; anteriorly very closely approximated, divergent to posterior margin; surface of metaventrite shiny with few micropunctures. Metacoxae shiny with few micropunctures; metacoxal lines distinct, broad apically, broadly curved with external surface convex, convergent anteriorly. Basal abdominal ventrites punctate, other surfaces of abdominal ventrites smooth, relatively shiny.

*Male genitalia*. Apex of median lobe in lateral aspect with apical blade narrow, curved, with apicoventral elongate, curved process, ventral margin subtruncate, proximal margin curved (Fig. 12a). Right lateral lobe in lateral aspect with apical segment longer than proximal segment; apical segment expanded medially, apex rounded (Fig. 12b); left lateral lobe with apical segment similar to right, but broader and more distinctly and prominently angulate along ventral margin (Fig. 12c).

*Variation*. Only a single male specimen examined.

*Sexual dimorphism*. Only a single male specimen examined.

#### Etymology.

This species is named *annae* after the author’s daughter, Annie Miller.

#### Distribution.

This species is known from one locality in Apure State, Venezuela (Fig. 30).

#### Habitat.

The habitat where the type series was collected is a sandy forest stream with large deposits of leaf pack along the margins.

#### Type material.

Holotype in MIZA, male labeled, “VENEZUELA: Apure State 4°58.838'N, 67°44.341'W, 95m Communidad Caño Gato, on Rio Sipapo: 16.i.2009; leg. Short, Miller, Camacho, Joly, & García VZ09-0116-01X; along stream/ SM0842863 KUNHM-ENT [barcode label]/ HOLOTYPE *Bidessonotus annae* Miller, 2016 [red label with black line border].” No other specimens examined.

### Bidessonotus josiahi

Taxon classificationAnimaliaColeopteraDytiscidae

Miller sp. n.

http://zoobank.org/49A491B8-9C3B-4D3C-868E-8F2F0B185DD0

Figs 6 , 13 , 31

#### Type locality.

Venezuela, Apure State, between “La Ye” and Bruzual, 7.644°N, 69.300°W.

#### Diagnosis.

Specimens of this species are brown with moderately distinctive maculae. The prosternal process is lanceolate and flat with the apex pointed. The female elytron is apicolaterally broadly lobed (Fig. 6b). The apical blade of the male median lobe is broad with the apicoventral angle developed into a rounded prominence, a broad apicodorsal tooth, the proximal margin straight without a tooth, and the ventral margin broadly pointed (Fig. 13a). The apical portions of the lateral lobes are broad with the apical segment of the right lateral lobe longer and broader with the apex very broadly rounded (Fig. 13b). The apical segment of the left lateral lobe is somewhat shorter and narrower than the right and the apex is very broadly truncate (Fig. 13c). The male genitalia are not similar to any other species. The proximal margin is nearly straight, without undulations or teeth. The apex is strongly obliquely truncate.

#### Description.

*Measurements*. TL = 2.2–2.3 mm, GW = 1.1–1.2 mm, PW = 0.9 mm, HW = 0.6 mm, EW = 0.3 mm, TL/GW = 2.0, HW/EW = 2.0. Body shape elongate, lateral outline discontinuous between pronotum and elytron.

*Coloration* (Fig. 6). Head orange. Pronotum yellow, dark along posterior margin. Elytron evenly dark brown except apex broadly pale (Fig. 6); without purplish iridescence in either sex. Ventral surfaces orange, darker orange laterally.

*Sculpture and structure*. Head with anterior clypeal margin evenly rounded; surface smooth and shiny, with few punctures medially; antennomeres III-X moderately broad, slightly asymmetrical. Pronotum widest near posterior angles, lateral margins evenly curved; basal striae strongly impressed, especially basally, broad, extending anteriorly more than halfway across surface; posterior margins distinctly undulate; surface overall slightly matte, but shiny, medial surface finely punctate. Elytron with lateral margins broadly curved; basal stria distinct, elongate, well impressed basally; surface of elytron covered with fine punctation, surface between punctures shiny. Prosternal process elongate, lanceolate, apically pointed, surface broadly convex throughout length. Metaventrite with distinctive carinae extending from medial apex of metaventrite process posteriorly to near posterior margin at anterior terminus of metacoxal lines; anteriorly very closely approximated, divergent to posterior margin; surface of metaventrite shiny with few micropunctures. Metacoxa shiny with few micropunctures; metacoxal lines distinct, broad, broadly curved with external surface convex, slightly convergent anteriorly. Basal abdominal ventrites punctate, other surfaces of abdominal ventrites smooth, relatively shiny.

*Male genitalia*. Apex of median lobe in lateral aspect with apical blade broad, with apicoventral rounded prominence, broad apicodorsal tooth, proximal margin straight, without tooth, ventral margin broadly pointed (Fig. 13a). Right lateral lobe in lateral aspect with apical segment about as long as proximal segment; apical segment apically slightly expanded, apex broadly rounded (Fig. 13b); left lateral lobe with apical segment shorter than basal segment, broad with apex very broadly truncate (Fig. 13c).

*Variation*. Specimens are variable in the extent of the pale markings on the elytron.

*Sexual dimorphism*. With typical *Bidessonotus* dimorphism. Males with apicolateral margin of elytron evenly curved (Fig. 6a); females apically with distinctive, subapical broad lobe (Fig. 6b).

#### Etymology.

This species is named *josiahi* after the author’s son, Josiah Miller.

#### Distribution.

The species is known from northern Venezuela (Fig. 31).

#### Habitat.

The type specimens were collected from a “lagoon.”

#### Type material.

Holotype in MIZA, male labeled, “VENEZUELA: Apure State 7.644°N, 69.300°W, 90, between “La Ye” & Bruzual 18.i.2009: Short, Camacho, & García: VZ09-0118-03X: lagoon/ SM0845741 KUNHM-ENT [barcode label]/ HOLOTYPE *Bidessonotus josiahi* Miller, 2016 [red label with red line border].” Paratypes 44; **Venezuela**: Anzoategui, Transect 1, 9.33°N, 64.196°W, 12 Aug 2009, Cordero, R (1, SEMC); Apure, between La Ye and Bruzual, 7.644°N, 69.300°W, 18 Jan 2009, Short, Camacho, Miller (6, SEMC); Guarico, N of Palenque, 9.113°N, 66.993°W, 08 Jan 2009, Short, Camacho, Garcia, Joly, Miller (3, SEMC); Monagas, El Guamo Reservoir, 10.102°N, 63.690°W, 28 Jan 2010, Short & Garcia (34, SEMC).

### Bidessonotus palecephalus

Taxon classificationAnimaliaColeopteraDytiscidae

Miller sp. n.

http://zoobank.org/4E6CCE60-C5C0-46B6-8229-6A30BB98E398

Figs 7 , 14 , 30

#### Type locality.

Venezuela, Guarico, N of Palenque, 9.113°N, 66.993°W.

#### Diagnosis.

This is a relatively pale brown species. The head color in specimens of *Bidessonotus palecephalus* is overall pale, unlike *Bidessonotus melanocephalus* which has the base of the head darkened. The prosternal process is flat and apically pointed. Females have the apicolateral elytral margins developed into a prominent lobe (Fig. 7b). Males of this species have the median lobe shape similar to those of *Bidessonotus melanocephalus* with a tooth medially on the dorsal margin near the apical base of the shaft as well as a tooth at each end of the distal margin (Fig. 14a), though the overall shapes are different in the two species. The lateral lobes are moderately similar in shape with the apical segments broad, but the right apical segment (Fig. 14b) is apically broadly rounded and the left apical segment is distinctly angulate along the ventral margin (Fig. 14c).

#### Description.

*Measurements*. TL = 1.9–2.0 mm, GW = 1.0 mm, PW = 0.8–0.9 mm, HW = 0.5–0.6 mm, EW = 0.3 mm, TL/GW = 1.8–2.0, HW/EW = 1.8–2.0. Body shape elongate oval, lateral outline discontinuous between pronotum and elytron.

*Coloration* (Fig. 7). Head, including all appendages and ventral surface, entirely pale yellow to yellow-orange. Pronotum yellow to yellow-orange. Elytron base color brown with large irregular yellow fasciae and maculae (Fig. 7); dorsal surfaces not purplish or iridescent in either sex. Ventral surfaces, including all legs and mouthparts, yellow, slightly darker along some sutures.

*Sculpture and structure*. Head with anterior clypeal margin slightly thickened, evenly rounded; surface smooth and shiny; antennomeres III-X moderately broad, slightly asymmetrical. Pronotum widest near posterior angles, lateral margins evenly curved; basal striae strongly impressed, broad, extending anteriorly more than halfway across surface; posterior margins distinctly undulate; surface overall slightly matte, but shiny, surface mediad of striae distinctly punctate. Elytron with lateral margins broadly curved; basal stria distinct, elongate, well impressed basally; surface of elytron covered with fine punctation, surface between punctures shiny. Prosternal process elongate, apically pointed, surface broadly convex throughout length. Metaventrite with distinctive carinae extending from medial apex of metaventrite process posteriorly to near posterior margin at anterior terminus of metacoxal lines; surface of metaventrite shiny with few micropunctures. Metacoxae shiny with few micropunctures; metacoxal lines distinct, broad, broadly curved with external surface convex, slightly convergent anteriorly. Basal abdominal ventrites punctate, other surfaces of abdominal ventrites smooth, relatively shiny.

*Male genitalia*. Apex of median lobe in lateral aspect with blade elongate, with apicoventral tooth, submedial broad tooth on distal margin and broad tooth along proximal margin, dorsal margin broad and obliquely truncate (Fig. 14a, d). Right lateral lobe in lateral aspect with apical segment about as long as proximal segment; apical segment apically broadly expanded, apex broadly rounded (Fig. 14b) left lateral lobe with apical segment very broad, apically very broadly rounded and with a distinct angulation along the ventral margin (Fig. 14c).

*Variation*. Specimens vary in the extent and intensity of the elytral fasciae and maculations.

*Sexual dimorphism*. With typical dimorphism found in *Bidessonotus*. Males with apicolateral margin of elytron evenly curved (Fig. 7a); females apically shortened, more abruptly rounded with distinctive, broadly rounded posterolateral expansion (Fig. 7b). Males with elytra shiny and smooth between punctures; females with elytra opaque and microreticulate.

#### Etymology.

The species is named *palecephalus* from the Greek words *pale*, meaning “pale,” and *cephalus*, meaning “head,” for the pale head of specimens in comparison with the somewhat similar species *Bidessonotus melanocephalus*.

#### Distribution.

The species is known from Apure and Guarico, Venezuela (Fig. 30).

#### Habitat.

Nothing is known of the habitat of this species.

#### Type material.

Holotype in MIZA, male labeled, “VENEZUELA: Guarico State 9.113°N, 66.993°W, 152m, Stream @ [sic] road crossing, N. of Palenque; 6.i.2009; leg. Short, García, Miller, Camacho, Joly VZ09-0108-03X; stream/ SEMC0854983 KUNHM-ENT [barcode label]/ HOLOTYPE *Bidessonotus palecephalus* Miller, 2016 [red label with black line border].” Paratypes, 31 total from the following localites: **Venezuela**: Apure, between La Ye and Bruzual, 7.644°N, 69.300°W, 18 Jan 2009, Short, Camacho, Miller (6, SEMC); Guarico, N of Palenque, 9.113°N, 66.993°W, 08 Jan 2009, Short, Camacho, Garcia, Joly, Miller (25, SEMC).

### Bidessonotus reductus

Taxon classificationAnimaliaColeopteraDytiscidae

Miller sp. n.

http://zoobank.org/6EEEE867-0465-44F6-BA7A-BC0E964097A8

Figs 8 , 15 , 31

#### Type locality.

Venezuela, Bolivar State, Gran Sabana, N Santa Elena, Rio Guara at Rt. 10, 04°37.362'N, 61°05.679'W.

#### Diagnosis.

Specimens of this species are small and pale (Fig. 8). The prosternal process is lanceolate and slightly sulcate with the apex pointed. The male genitalia in the species are quite unique. The apical blade of the male median lobe is narrowed and elongate-triangular with the dorsal margin sharply pointed (Fig. 15a). The right lateral lobe has the apical portion very large and broad with a very broad expansion along the ventral margin (Fig. 15b). The right lateral lobe has the apical portion somewhat smaller relative to the basal segment but also broad and distinctly expanded along the ventral margin (Fig. 15c). Females have the apicolateral elytral margins unmodified.

#### Description.

*Measurements*. TL = 1.8–2.0 mm, GW = 0.9–1.0 mm, PW = 0.8 mm, HW = 0.5 mm, EW = 0.3 mm, TL/GW = 2.0, HW/EW = 1.7–1.8. Body shape elongate oval, lateral outline discontinuous between pronotum and elytron.

*Coloration* (Fig. 15). Head orange yellow. Pronotum yellow. Elytron base color light brown with diffuse, irregular pale fasciae, oriented more-or-less longitudinally, apex pale; female with indistinct purplish iridescence. Ventral surfaces yellow on all surfaces.

*Sculpture and structure*. Head with anterior clypeal margin evenly rounded; surface smooth and shiny, with few micropunctures medially; antennomeres III-X moderately broad, slightly asymmetrical. Pronotum widest near posterior angles, lateral margins evenly curved; basal striae distinctly impressed, especially basally, elongate, extending anteriorly more than halfway across surface; posterior margins distinctly undulate; surface shiny, medial surface finely punctate. Elytron with lateral margins broadly curved; basal stria distinct, elongate, well impressed; surface of elytron covered with fine punctation, surface between punctures somewhat shiny but microreticulate. Prosternal process moderately broad, broadly oval, apex rounded, surface broadly convex throughout length. Metaventrite with distinctive carinae extending from medial apex of metaventrite process posteriorly to near posterior margin at anterior terminus of metacoxal lines; anteriorly very closely approximated, strongly divergent to posterior margin; surface of metaventrite shiny, impunctate. Metacoxae shiny, nearly impunctate; metacoxal lines distinct, broadly separated, anteriorly slightly divergent. Abdominal ventrites smooth, relatively shiny.

*Male genitalia*. Apex of median lobe in lateral aspect with blade reduced, slender, sharply pointed at dorsal margin (Fig. 15a). Right lateral lobe in lateral aspect with apical segment much longer than proximal segment; apical segment apically very broadly expanded medially, apex broadly rounded (Fig. 15b); left lateral lobe with apical segment shorter than basal segment, broad with apex truncate and with distinct expansion along ventral margin (Fig. 15c).

*Variation*. The two specimens vary somwhat in the extent and intensity of the pale elytral areas.

*Sexual dimorphism*. Specimens of this species with typical *Bidessonotus* sexual dimorphism and female more matte dorsally than male. Without other evident dimorphisms.

#### Etymology.

This species is named *reductus*, Latin for “reduced,” for the relatively small apical blade of the male median lobe.

#### Distribution.

Known only from the Gran Sabana, Bolivar State, Venezuela (Fig. 31).

#### Habitat.

Specimens have been collected from “marshy areas.”

#### Type material.

Holotype in MIZA, male labeled, “VENEZUELA: Bolivar State 04°37.362'N, 61°05.679'W, 876 m Gran Sabana, N. Santa Elena Rio Guara at Rt. 10. 17.VII.2010 Leg. Short, Tellez & Arias marshy area; VZ10-0717-02A/ SEMC0908509 KUNHM-ENT [barcode label]/ HOLOTYPE *Bidessonotus reductus* Miller, 2016 [red label with double black line border].” Paratype, 1 female, **Venezuela**, Bolivar State, 04°41.878'N, 61°04.246'W, 815m, Gran Sabana, N Santa Elena, marshy area along Rt 10, Short, Tellez and Camacho, legs. 17 Jul 2010, VZ10-0717-03A, SEMC0908642.

### Bidessonotus septimus

Taxon classificationAnimaliaColeopteraDytiscidae

Miller sp. n.

http://zoobank.org/7D5AC872-4A0C-4351-B079-64132D28A150

Figs 9 , 16 , 32

#### Type locality.

Venezuela, Apure, Bruzual, edge of town, 8.042°N, 69.342°W.

#### Diagnosis.

Specimens of this species are medium-sized and dark with moderately well-developed, but somewhat diffuse maculae. The prosternal process is moderately broad, sulcate and apically pointed. The male genitalia are characteristic with the apical blade of the median lobe broad with a moderately well-developed apicoventral triangular prominence, the dorsal margin rounded, and the proximal margin oblique and distinctly undulate (Fig. 16a). The lateral lobes have the apical segments rather different in shape with the right lateral lobe apically elongate, apically rounded and expanded along the ventral margin (Fig. 16b). The left lateral lobe has the apical segment short, broad and apically distinctly concave making an obliquely bilobed margin (Fig. 16b). This shape is somewhat similar to that of *Bidessonotus dubius* (Fig. 20), but is more elongate with the dorsal margin distinctly rounded.

#### Description.

*Measurements*. TL = 2.0 mm, GW = 1.0 mm, PW = 0.8 mm, HW = 0.6 mm, EW = 0.2 mm, TL/GW = 2.0, HW/EW = 2.3. Body shape elongate, lateral outline discontinuous between pronotum and elytron.

*Coloration* (Fig. 9). Head yellow. Pronotum yellow, darker along posterior margin. Elytron base color dark brown with diffuse, irregular pale maculae antero- and mediolaterally, apex pale (Fig. 9); purplish iridescence not evident in male specimen. Ventral surfaces yellow orange on all surfaces.

*Sculpture and structure*. Head with anterior clypeal margin evenly rounded; surface smooth and shiny, with few micropunctures medially; antennomeres III-X moderately broad, slightly asymmetrical. Pronotum widest near posterior angles, lateral margins evenly curved; basal striae strongly impressed, especially basally, elongate, extending anteriorly more than halfway across surface; posterior margins distinctly undulate; surface shiny, medial surface finely punctate. Elytron with lateral margins broadly curved; basal stria distinct, elongate, well impressed; surface of elytron covered with fine punctation, surface between punctures matte, microreticulate. Prosternal process narrow, elongate, lanceolate, apex pointed, surface broadly convex throughout length. Metaventrite with carinae extending from medial apex of metaventrite process posteriorly, effaced and indistinct for much of length, marked mainly by smooth longitudinal area; anteriorly very closely approximated, strongly divergent to posterior margin; surface of metaventrite shiny, impunctate. Metacoxae shiny, nearly impunctate; metacoxal lines distinct, moderately separated, subparallel, slightly curved anteriorly forming slight lateral bulge. Abdominal ventrites smooth, relatively shiny.

*Male genitalia*. Apex of median lobe in lateral aspect with blade broad, with apicoventral tooth, ventral tooth near apical base of shaft, angulate, oblique proximal margin, dorsal margin rounded, produced (Fig. 16a). Right lateral lobe in lateral aspect with apical segment about as long as proximal segment; apical segment apically slightly expanded, apex rounded (Fig. 16b); left lateral lobe short, broad, apically obliquely concave making margin obliquely and broadly bilobed (Fig. 16c).

*Variation*. Only a single specimen was examined.

*Sexual dimorphism*. Only a single specimen was examined.

#### Etymology.

This species is named *septimus*, Latin for “seventh,” since there are seven species described in *Bidessonotus* herein.

#### Distribution.

The single specimen was found in Apure State, Venezuela.

#### Habitat.

The holotype was collected from a “large marsh.”

#### Type material.

Holotype in MIZA, male labeled, “VENEZUELA: Apure State 8°2.534'N, 69°20.530' 83m edge of Bruzual; 18.i.2009 leg, Short, García, Camacho VZ09-0118-04X; large marsh/ SM0844586 KUNHM-ENT [barcode label]/ HOLOTYPE *Bidessonotus septimus* Miller, 2016 [red label with black line border].”

### Bidessonotus spinosus

Taxon classificationAnimaliaColeopteraDytiscidae

Miller sp. n.

http://zoobank.org/17890E01-A262-4AF8-B28C-D991C756071E

Figs 10 , 17 , 30

#### Type locality.

Venezuela, Bolivar, Gran Sabana, Rio Aponwao at Rt 10, 5.847°N, 61.467°W.

#### Diagnosis.

Individuals have brown elytra with diffuse, poorly defined paler regions. The prosternal process is broadly oval, apically rounded and not sulcate. The female elytron has the apicolateral margin developed into a distinctive spine (Fig. 10b). The apical blade of the male median lobe is very broad with a spinous process at the anteroventral angle, a long, spinous process medially on the distal margin, and the dorsal margin irregularly subtruncate (Fig. 17a). The right lateral lobe has the apical segment broadly expanded along the ventral margin and about as long as the basal segment (Fig. 17b). The left lateral segment is shorter and broader with a distinct, rounded angulation along the ventral margin (Fig. 17c). The male genitalia (Fig. 17a,d) are similar to *Bidessonotus truncatus* (Fig. 28a,d) in having a distinctive spine along the apical margin. The apical blade is much broader and shorter with the proximal margin irregularly toothed (Fig. 17a,d).

**Figures 19–29.**
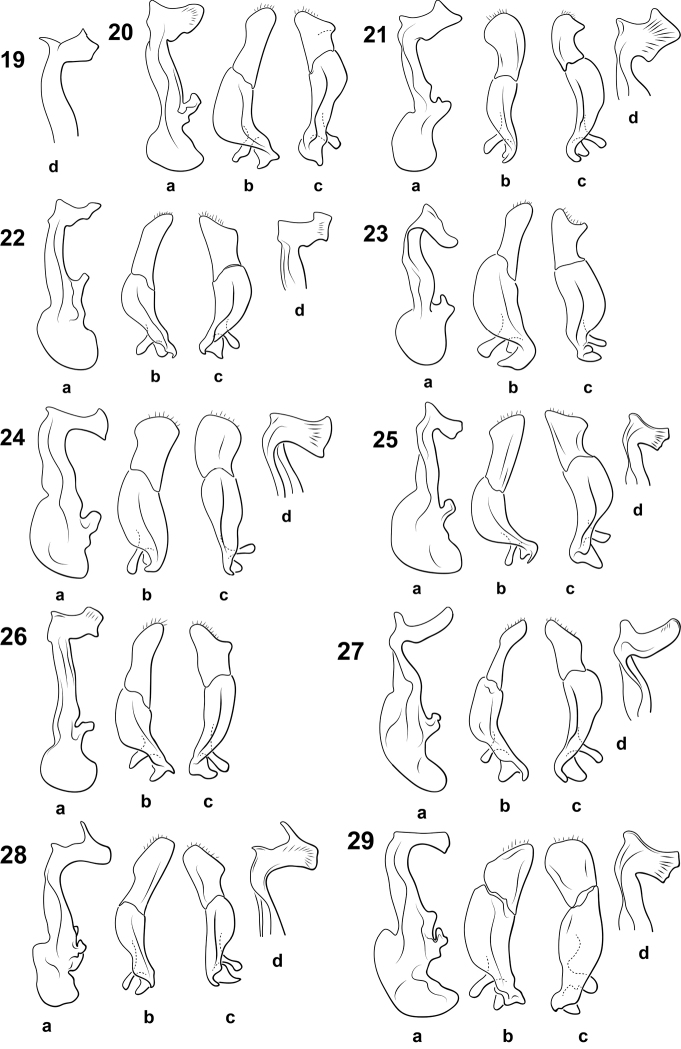
*Bidessonotus* species, male genitalia; **A** median lobe, right lateral aspect **B** right lateral lobe, right lateral aspect **C** left lateral lobe, apical segment, left lateral aspect **D** median lobe apex, oblique right lateral aspect **19** *Bidessonotus bicolor* (redrawn from Guignot, 1957) **20** *Bidessonotus dubius* **21** *Bidessonotus melanocephalus* **22** *Bidessonotus obtusatus* **23** *Bidessonotus otrerus* **24** *Bidessonotus paludicolus* **25** *Bidessonotus ploterus* **26** *Bidessonotus rubellus* **27** *Bidessonotus tibialis* **28** *Bidessonotus truncatus* **29** *Bidessonotus vicinus*.

#### Description.

*Measurements*. TL = 2.0 mm, GW = 1.0 mm, PW = 0.8–0.9 mm, HW = 0.6 mm, EW = 0.3 mm, TL/GW = 2.0, HW/EW = 1.7–1.8. Body shape elongate oval, lateral outline discontinuous between pronotum and elytron.

*Coloration* (Fig. 10). Head orange. Pronotum entirely yellow. Elytron base color brown with broad, diffuse, somewhat transverse slightly paler regions, margin of regions vague and indistinct (Fig. 10); without purplish dorsal iridescence. Prosternal surface yellow; other thoracic ventrites orange except metacoxa darker orange.

*Sculpture and structure*. Head with anterior clypeal margin slightly thickened laterally, broadly rounded; surface smooth and shiny; antennomeres III-X moderately broad, slightly asymmetrical. Pronotum widest near posterior angles, lateral margins evenly curved; basal striae moderately impressed, extending anteriorly more than halfway across surface; posterior margins distinctly undulate; surface overall shiny, surface mediad of striae slightly punctate. Elytron with lateral margins broadly curved; basal stria distinct, moderately elongate, well impressed basally; surface of elytron covered with punctation, surface between punctures shiny but with distinctive microreticulation. Prosternal process elongate, lanceolate, apically pointed, surface broadly convex throughout length. Metaventrite with carinae extending from medial apex of metaventrite process posteriorly to posterior margin at anterior terminus of metacoxal lines; lines narrowly separated anteriorly, slightly divergent posteriorly and somewhat effaced; surface of metaventrite shiny with few micropunctures. Metacoxa shiny with few micropunctures; metacoxal lines distinct, nearly parallel, width slightly increased near anterior margin making lines slightly undulate anteriorly. Basal abdominal ventrites punctate, other surfaces of abdominal ventrites smooth, relatively shiny.

*Male genitalia*. Apex of median lobe in lateral aspect with apical blade broad, with apicoventral sharp tooth, long spinous apicodorsal tooth, dorsal margin broadly subtruncate, proximal margin irregular (Fig. 17a). Right lateral lobe in lateral aspect with apical segment about as long as proximal segment; apical segment very broadly expanded with rounded lobe along ventral margin, apex broadly rounded (Fig. 17b); left lateral lobe lateral aspect with apical segment broader and shorter than right with distinct, angulate expansion along ventral margin (Fig. 17c).

*Variation*. The two specimens vary in the extent and intensity of the elytral fasciae and maculations.

*Sexual dimorphism*. With typical sexual dimorphism for *Bidessonotus*. Male with anteroapical margin of elytron evenly curved; female with anteroapical margin produced into small spine (Fig. 10b).

#### Etymology.

This species is named *spinosus*, Latin for “thorny,” for the long apical spine on the apex of the male median lobe.

#### Distribution.

**Figures 30–31.**
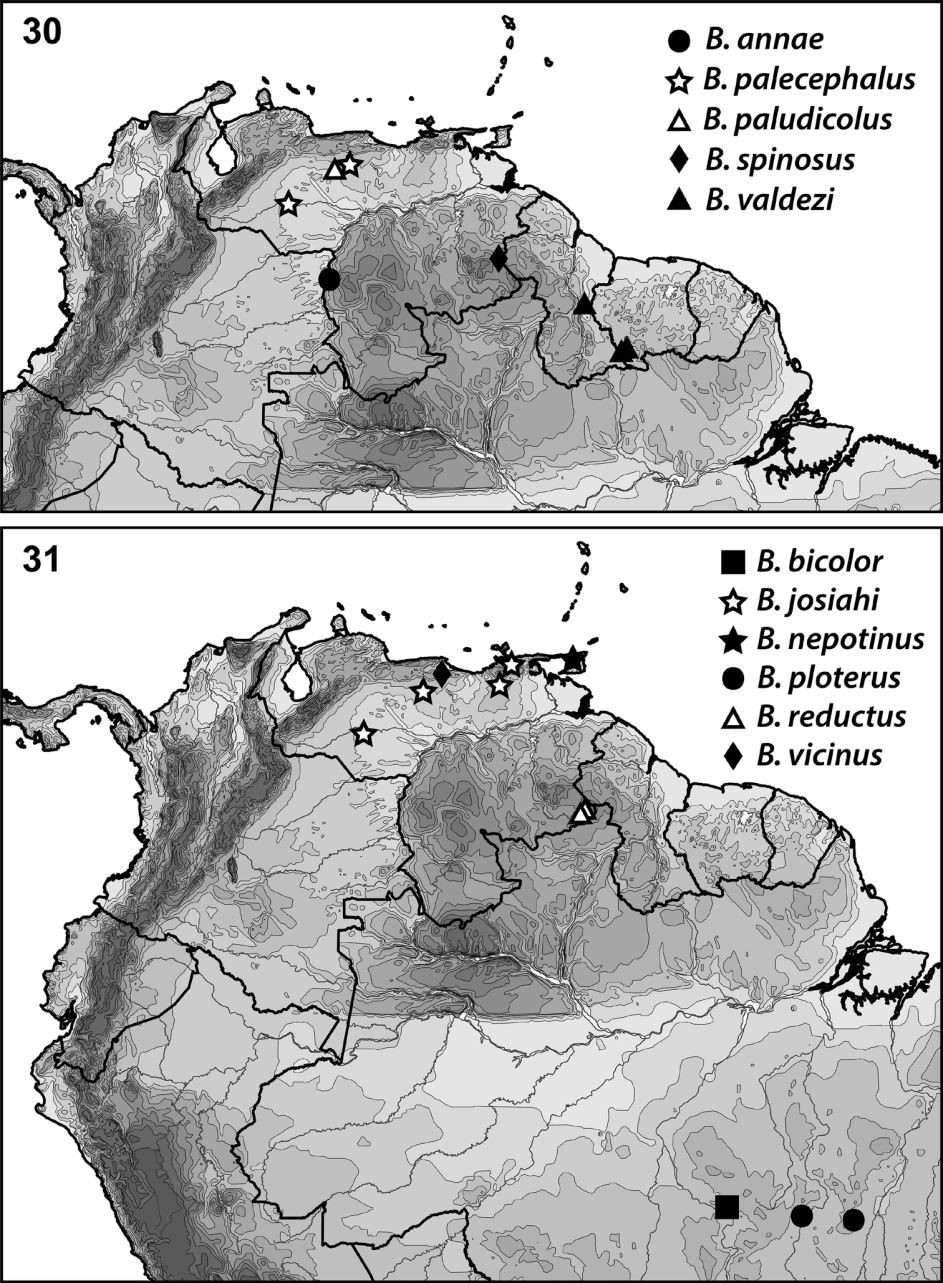
*Bidessonotus* species, South American distributions.

Known from one locality in Bolivar State, Venezuela (Fig. 30).

#### Habitat.

Specimens were collected from a “small vegetated pool” and a “small streamlet.”

#### Type material.

Holotype in MIZA, male labeled, “VENEZUELA: Bolivar State 6°50'49.2"N, 61°28.2'2.4"W, 1340m Rio Agonwao @ [sic] Hwy 10 31.vii.2008; leg. A.Short, M. García AS-08-060a; small vegetated pool/ SM0827643 KUNHM-ENT/ HOLOTYPE *Bidessonotus spinosus* Miller, 2016 [red label with red line border].” Paratypes, 1, **Venezuela**: Bolivar State, 6°50'49.2"N, 61°28.2'2.4"W, Rio Aponwao, 1340m, 31 Jul 2008, small streamlet, A. Short, M. García, legs., AS-08-060b, SM0830163.

### Bidessonotus valdezi

Taxon classificationAnimaliaColeopteraDytiscidae

Miller sp. n.

http://zoobank.org/5837C9C7-606B-4F1B-9082-59EED7A0610F

Figs 11 , 18 , 30

#### Type locality.

Guyana, Region 6, Upper Berbice, ca 1km S Basecamp 1, 4°09.289'N, 58°12.274'W.

#### Diagnosis.

Specimens of *Bidessonotus valdezi* are relatively pale brown with the elytral maculae indistinct and vague. The prosternal process is lanceolate, shallowly sulcate and apically pointed. The apical blade of the male median lobe is broad with a moderately developed apicoventral triangular prominence, the distal margin sublinear, the proximal margin obliquely linear, and the dorsal margin narrowly truncate (Fig. 18a). The lateral lobes are distinctive, the right lateral lobe has the apical segment long, slender and apically rounded (Fig. 18a). The left lateral lobe is shorter, broader with the lateral margins evenly convergent to a rounded apex (Fig. 18c). Females have the apicolateral margin of the elytron unmodified. The male median lobe is somewhat similar in shape to the species *Bidessonotus peregrinus* J. Balfour-Browne, but that species (known from Panama) is smaller (<1.8 mm) and there are some differences in the male genitalia. In *Bidessonotus peregrinus* the apical blade of the median lobe is much more narrow and the lateral lobes are broader with the apical segments on each side differently shaped between the two species. Specimens of *Bidessonotus valdezi* are not dorsally iridescent, whereas females of *Bidessonotus peregrinus* usually are.

#### Description.

*Measurements*. TL = 1.8–1.9 mm, GW = 0.9 mm, PW = 0.7 mm, HW = 0.5 mm, EW = 0.3 mm, TL/GW = 2.0–2.1, HW/EW = 1.8. Body shape elongate oval, lateral outline discontinuous between pronotum and elytron.

*Coloration* (Fig. 11). Head, including all appendages and ventral surface, entirely pale yellow to yellow-orange. Pronotum entirely yellow. Elytron base color brown with broad, diffuse, somewhat transverse slightly paler regions, margin of regions vague and indistinct (Fig. 11); without purplish iridescence. Prosternal surface yellow; other thoracic ventrites orange except metacoxa darker orange.

*Sculpture and structure*. Head with anterior clypeal margin slightly thickened laterally, broadly rounded; surface smooth and shiny; antennomeres III-X moderately broad, slightly asymmetrical. Pronotum widest near posterior angles, lateral margins evenly curved; basal striae moderately impressed, extending anteriorly more than halfway across surface; posterior margins distinctly undulate; surface overall shiny, surface mediad of striae slightly punctate. Elytron with lateral margins broadly curved; basal stria distinct, moderately elongate, well impressed basally; surface of elytron covered with punctation, surface between punctures shiny but with distinctive microreticulation. Prosternal process elongate, lanceolate, apically pointed, surface broadly convex throughout length. Metaventrite with carinae extending from medial apex of metaventrite process posteriorly to posterior margin at anterior terminus of metacoxal lines; lines narrowly separated anteriorly, slightly divergent posteriorly and somewhat effaced; surface of metaventrite shiny with few micropunctures. Metacoxae shiny with few micropunctures; metacoxal lines distinct, nearly parallel, width slightly increased near anterior margin making lines slightly undulate anteriorly. Basal abdominal ventrites punctate, other surfaces of abdominal ventrites smooth, relatively shiny.

*Male genitalia*. Apex of median lobe in lateral aspect with apical blade moderately broad, with anteroventral triangular prominence, dorsal margin truncate, proximal margin obliquely curved (Fig. 11a). Lateral lobe in lateral aspect with apical segment about as long as proximal segment; apical segment relatively narrow, apex rounded (Fig. 11b).

*Variation*. Specimens vary in the extent and intensity of the elytral fasciae and maculations. In some specimens the lighter regions of the elytra are somewhat more intensely pale and slightly more distinctly defined.

*Sexual dimorphism*. With typical sexual dimorphisms for *Bidessonotus* species. Females with dorsal surface more matte than in males.

#### Etymology.

The species is named *valdezi* after the good friend of the author and eminent biologist, Dr. Ernest Valdez.

#### Distribution.

Known from Guyana and Suriname (Fig. 30).

#### Habitat.

Specimens have been found in “muddy detrital pools” in a drying creek bed,“detritus pools” in a dry creek bed, and a “pooled up creek.”

#### Type material.

Holotype in CSBD, male labeled, “GUAYANA: Region 6 4°09.289'N, 58°12.274'W, 108m Upper Berbice, ca. 1 km S. Basecamp 1 detrius pools in dry creekbed leg. Short, Salisbury, La Cruz 26.ix.2014; GY14-0825-01D/ SECM1358746 KUNHM-ENT [barcode label]/ HOLOTYPE *Bidessonotus valdezi* Miller, 2016 [red label with black line border].” paratypes 12, **Guyana**: Region 6, Upper Berbice, Basecamp 1, 4°09.289'N, 58°12.274'W, 72m, muddy detrital pools in drying creekbed near camp, Short, Salisburg, La Cruz, legs., 21 Sep 2014, GY14-0821-02A (2, SEMC); Region 6, Upper Berbice, ca 1km S Basecamp 1, 4°09.241'N, 58°10.627'W, detritus pools in dry creekbed, Short, Salisbury, La Cruz, legs, 26 Sep 2014, GY14-0925-010 (7, SEMC); Region 6, Upper Berbice, 3km W Basecamp 1, 4°09.297'N, 58°00.431'W, pooled up creek, Short, Salisbury, La Cruz, legs, GY14-0923-01A (3, SEMC). **Suriname**: Sipaliwini, Camp 2, on Sipaliwini river, 2.182°N, 56.787°W, 28 Aug 2010, Short & Kadosoe (5, SEMC); Sipaliwini, Camp 3, Wehepai, 2.362°N, 56.697°W, 03 Sep 2010, Short & Kadosoe (16, SEMC).

### Bidessonotus bicolor

Taxon classificationAnimaliaColeopteraDytiscidae

Guignot, 1957

Figs 19 , 31

Bidessonotus bicolor Guignot, 1957: 36; Young 1969: 2; 1990: 378; Biström 1988: 18; Nilsson 2016: 99.

#### Diagnosis.

*Bidessonotus bicolor* is inadequately known and a diagnosis is difficult to establish. The apical blade of the male median lobe is relatively small with a spinous process at the apicoventral angle, a triangular prominence medially on the distal margin, and a pointed dorsal apex (Fig. 19).

#### Discussion.

The type was not found and no other specimens were examined by Young (1990), who thought the species might be based on a teneral specimen of *Bidessonotus melanocephalus*. No specimens were examined for this study either.

#### Distribution.

Known only from the type locality, Brazil, Pará, Cachimbo (Fig. 31).

### Bidessonotus dubius

Taxon classificationAnimaliaColeopteraDytiscidae

Young, 1990

Figs 20 , 33

Bidessonotus dubius Young, 1990a: 364; Nilsson 2016: 99.

#### Diagnosis.

Specimens of *Bidessonotus dubius* are moderately darkly colored with fairly well defined pale maculae on the elytra. The prosternal process is narrow and not or slightly sulcate and apically pointed. The apical blade of the male median lobe is diagnostic with a weakly developed apicoventral prominence and the dorsal apex broadly rounded with an undulating proximal margin (Fig. 20a). The male right lateral lobe has the apical segment relatively narrow and as long as the basal segment (Fig. 20b). The left lateral lobe has the apical segment shorter with the apex broadly obliquely truncate with a small distinctive lobe along the ventral margin (Fig. 20c).

#### Discussion.

Young (1990) thought this species is similar to *Bidessonotus obtusatus*. The species is one of the most common in northern South America and is found especially in marshes and ponds.

#### Distribution.

Known from throughout northern South America (Fig. 33). Young (1990) reported *Bidessonotus dubius* from numerous sites in Brazil, French Guyana, Suriname and Venezuela. Examined specimens include the following: **Guyana**: Region 9, Karanambo, 3.749°N, 59.299°W, 02 Apr 1994, PJ Spangler (2, USNM); Region 9, Karanambo, Simoni Lake, 3.749°N, 59.299°W, 02 Apr 1994, PJ Spangler (2, USNM); Region 9, Parabara, at N. edge of village, 2.095°N, 59.239°W, 03 Nov 2013, Short, Isaacs, Salisbury (2, SEMC); Pirara Ranch, Pirara River, 3.535°N, 59.675°W, 24 Apr 1995, Spangler & Perry (6, USNM). **Suriname**: Para, Paramaribo, 25 km S, 5.578°N, 55.192°W, 12 Jul 1969, PJ Spangler (1, USNM); Suriname: Saramacca, Sidiredjo, 1 km E, 5.830°N, 55.533°W, 5 Mar 2012, Short & Kadosoe (11, SEMC). **Venezuela**: Amazonas, Puerto Ayacucho, approx 15 km S, nr. Campamento Canturama, 5.510°N, 67.601°W, 08 Aug 2008, AE Short (1, SEMC); Apure, between La Ye and Bruzual, 7.644°N, 69.300°W, 18 Jan 2009, Short, Camacho, Miller (8, SEMC); Apure, Bruzual, edge of town, 8.042°N, 69.342°W, 18 Jan 2009, Short, Camacho, Miller (7, MIZA); Apure, Mantecal, approx 10 km W, side road, 7.621°N, 69.061°W, 18 Jan 2009, Short, Camacho, Miller (32, SEMC); Barinas, SW of Batatuy, 8.170°N, 70.864°W, 25 Jan 2012, Short, Arias, Gustafson (1, SEMC); Barinas, Ciudad Bolivia, approx 13 km SE, large Hacienda, 8.323°N, 70.470°W, 25 Jan 2012, Short, Arias, Gustafson (128, SEMC); Barinas, Ciudad Bolivia, approx 20 km S, small stream, 8.282°N, 70.397°W, 25 Jan 2012, Short, Arias, Gustafson (5, SEMC); Barinas, E Los Pasitos, 8.474°N, 70.536°W, 14 Jul 2009, Short, Camacho, Inciarte, Garcia, Gustafson, Shepard, Sites (11, SEMC); Bolivar, E Tumeremo, on road to Bochinche, 7.384°N, 61.325°W, 13 Jul 2010, Short, Arias, Tellez (7, SEMC); Cojedes, El Baul, 5 km S, large marsh, 8.900°N, 68.321°W, 21 Jan 2012, Short, Arias, Gustafson (13, SEMC); Cojedes, El Pao at Embalsa El Pao, next to main church, 9.636°N, 68.126°W, 21 Jan 2012, Short, Arias, Gustafson (3, SEMC); Delta Amacuro, Between Tucupita & Los Guires, 9.175°N, 61.910°W, 3 Feb 2010, Short & Garcia (7, SEMC); Delta Amacuro, between Tucupita & Temblador, small pond along road, 8.773°N, 62.238°W, 3 Feb 2010, Short, Garcia, Joly (3, SEMC); Delta Amacuro, Transect 3, 9.118°N, 61.959°W, 27 Aug 2009, R Cordero (1, SEMC); Delta Amacuro, Transect 3, 9.188°N, 61.883°W, 24 Aug 2009, R Cordero (2, SEMC); Guarico, between Palenque & Las Mercedes, 9.0631°N, 66.610°W, 4 Jul 2010, Short, Camacho, Tellez (9, SEMC); Guarico, 32 km SW Calabozo, 8.664°N, 67.552°W, 11 Feb 1969, PJ Spangler (85, USNM); Guarico, Hato Masaguaral, 40 km S of Calabozo, 8.566°N, 67.583°W, 5 Mar 1986, Spangler (628, USNM); Guarico, Hato Masaguaral, 44 km S of Calabozo, Gate Lagoon, 8.566°N, 67.583°W, 6 Mar 1986, Spangler (97, USNM); Guarico, Las Mercedes, approx 65 km S, 8.528°N, 66.376°W, 06 Jul 2010, Short, Arias, Camacho, Tellez (41, SEMC); Guarico, San Fernando, 7.942°N, 67.480°W, 12 Feb 1969, PJ Spangler (31, USNM); Guarico, Santa Rita, 2.6 km W, 8.121°N, 66.278°W, 6 Jul 2010, Short & Tellez (1, SEMC); Monagas, Maturin, S of, river crossing, 9.609°N, 63.138°W, 02 Feb 2010, Short & Garcia (33, SEMC); Monagas, Rio Azagua, 10.014°N, 63.142°W, 31 Jan 2010, Joly T, Louis J (23, MIZA); Monagas, S of Maturin, Morichal at road crossing, 9.273°N, 62.937°W, 2 Feb 2010, Short, Garcia, Joly (3, SEMC); Portuguesa, Guanare, N of, Rio Guanare, 9.041°N, 69.816°W, 19 Jan 2009, Garcia, Mauricio (6, MIZA); Sucre, El Pilar, approx 5 km SE, 10.523°N, 63.117°W, 29 Jan 2010, Short & Garcia (45, SEMC); Sucre, Finca Vuelta Larga, 10.501°N, 63.103°W, 29 Jan 2010, Short, Garcia, Joly (10, SEMC); Tachira, La Pedrera, 10 km E, Mata de Limon, small lagoon on finca, 7.502°N, 71.488°W, 26 Jan 2012, Short, Arias, Gustafson (17, SEMC); Trujillo, Agua Viva, NE, 9.629°N, 70.587°W, 29 Jan 2012, Short & Gustafson (1, SEMC); Trujillo, Granados, approx 3 km SW, 9.376°N, 70.818°W, 28 Jan 2012, Short, Arias, Gustafson (2, SEMC); Trujillo, La Cieba, approx 10 km E, by cemetery, 9.4750°N, 70.955°W, 28 Jan 2012, Short, Arias, Gustafson (2, SEMC); Zulia, Puente del Zulia, lagoon on finca, 8.551°N, 72.336°W, 27 Jan 2012, Short, Arias, Gustafson (3, SEMC).

**Figure 32.**
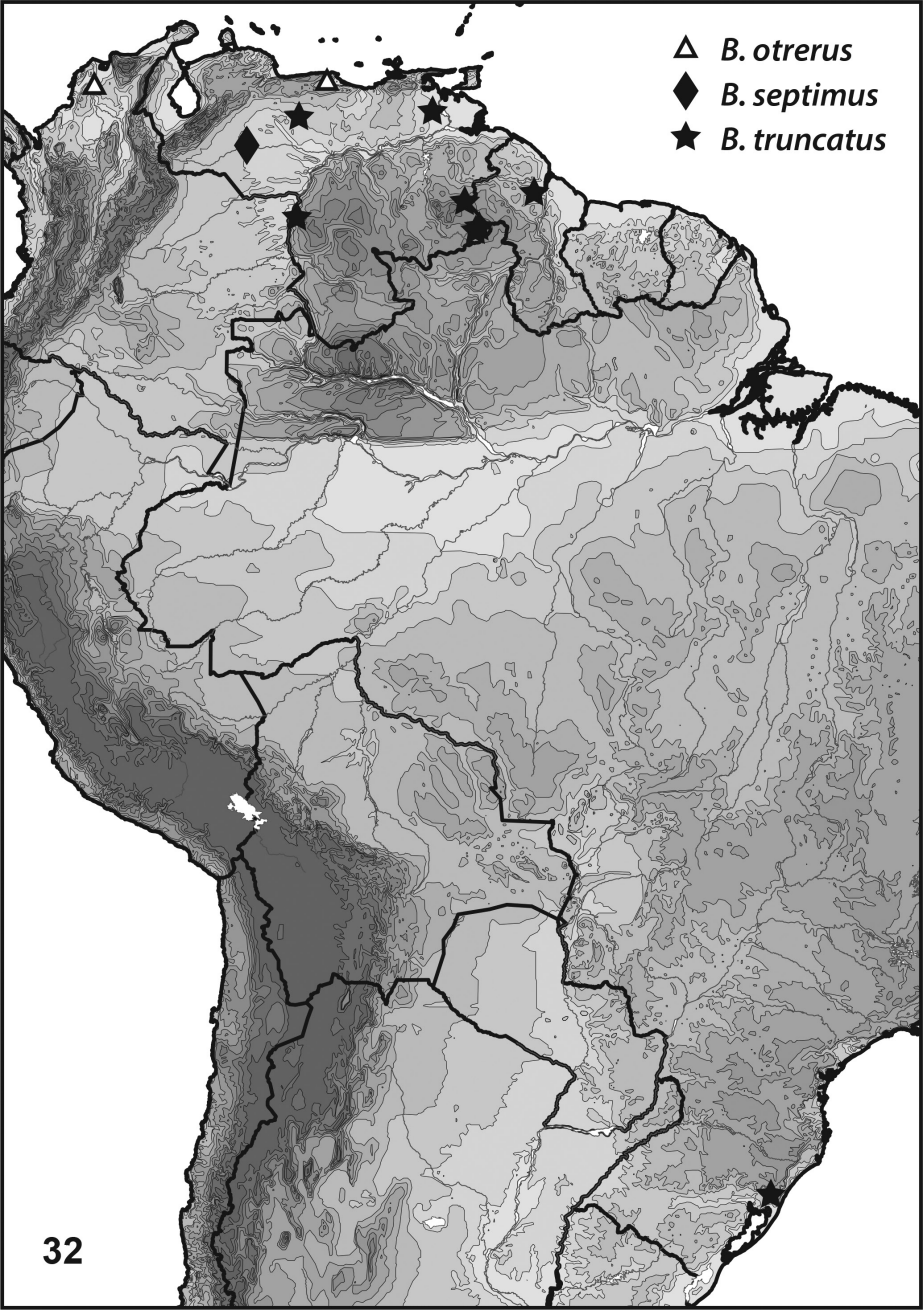
*Bidessonotus* species, South American distributions.

**Figures 33–34.**
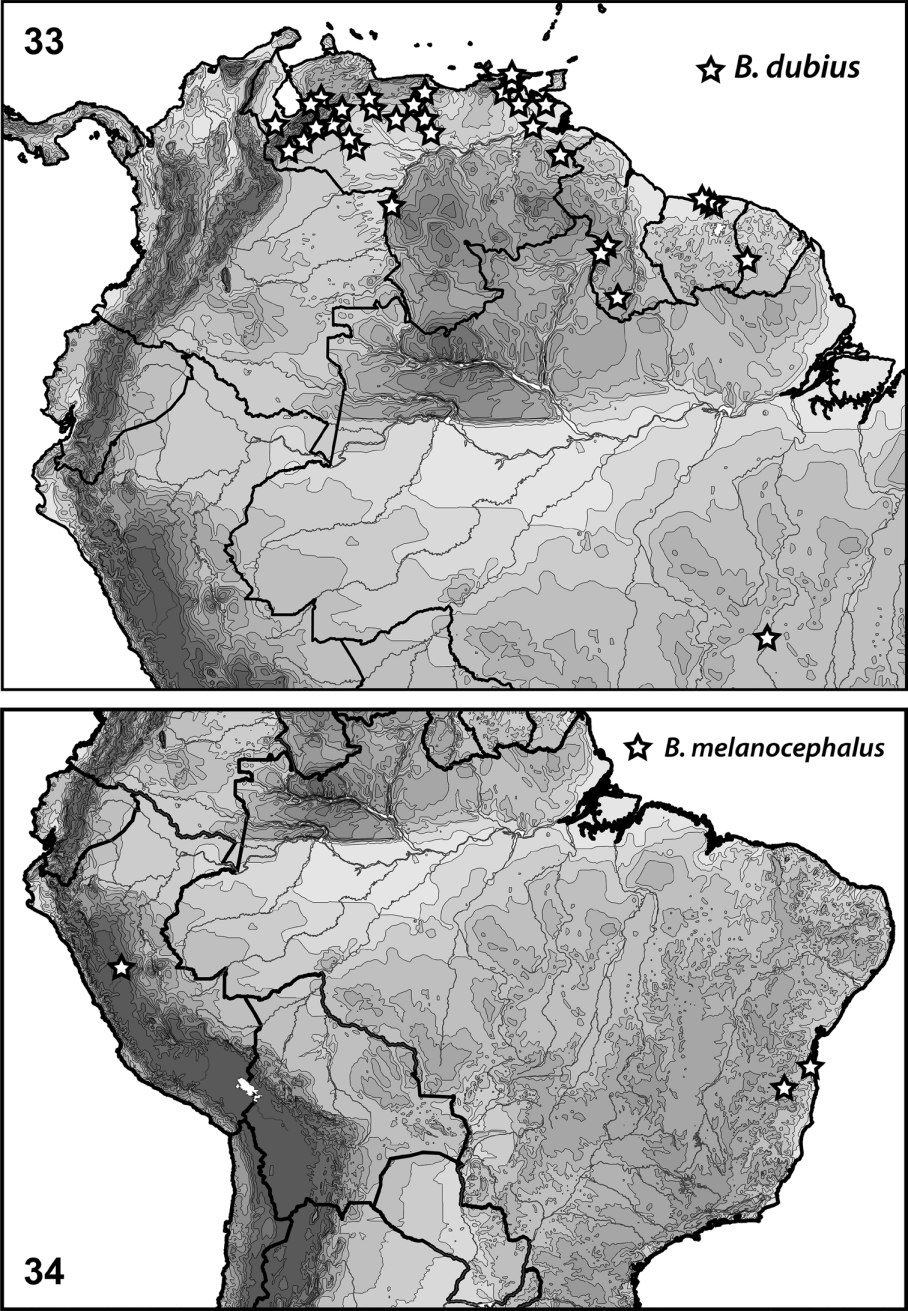
*Bidessonotus* species, South American distributions.

### Bidessonotus melanocephalus

Taxon classificationAnimaliaColeopteraDytiscidae

Régimbart, 1895

Figs 21 , 34

Bidessonotus melanocephalus Régimbart, 1895: 332; J. Balfour-Browne 1947: 443; Young 1969: 2; 1990: 375; Biström 1988: 18; Nilsson 2016: 99.
Bidessus (Bidessonotus) melanocephalus , Zimmermann 1920: 62.
Bidessus melanocephalus , Blackwelder 1944: 76.

#### Diagnosis.

Specimens of *Bidessonotus melanocephalus* are darkly colored with the posterior surface of the head and the ventral surfaces dark brown. The prosternal process is lanceolate but not sulcate. The apical blade of the male median lobe is very broad with very well developed apicoventral and apicodorsal teeth and a broad tooth near the medial end of the proximal margin (Fig. 21a,d). The right lateral lobe has the apical segment apically broadly rounded and expanded along the ventral margin (Fig. 21b). The apical segment of the left lateral lobe is short, broad and curve and distinctly pointed along the dorsal margin (Fig. 21c).

#### Discussion.

Young (1990) compared this species with *Bidessonotus inconspicuus*. This is one of only a couple *Bidessonotus* species known from central and southern South America.

#### Distribution.

Known from Brazil and Peru (Fig. 34, Young 1990).

### Bidessonotus nepotinus

Taxon classificationAnimaliaColeopteraDytiscidae

J. Balfour-Browne, 1947

Fig. 31

Bidessonotus nepotinus J.Balfour-Browne, 1947: 442; Young 1969: 2; 1990: 364; Biström 1988: 18; Nilsson 2016: 99.

#### Diagnosis.

Known only from a pair of females which are moderately darkly colored with three indistinct transverse maculae. The apex of the prosternal process is lanceolate. Given the absence of known males, a definitive diagnosis is difficult to establish.

#### Discussion.

*Bidessonotus nepotinus* is known only from two female specimens (Balfour-Browne 1947; Young 1990). Balfour-Browne (1947) thought the species is close to or identical with *Bidessonotus obtusatus*.

#### Distribution.

*Bidessonotus nepotinus* is known only from Trinidad (Fig. 31).

### Bidessonotus obtusatus

Taxon classificationAnimaliaColeopteraDytiscidae

Régimbart, 1895

**Figures 35–36.**
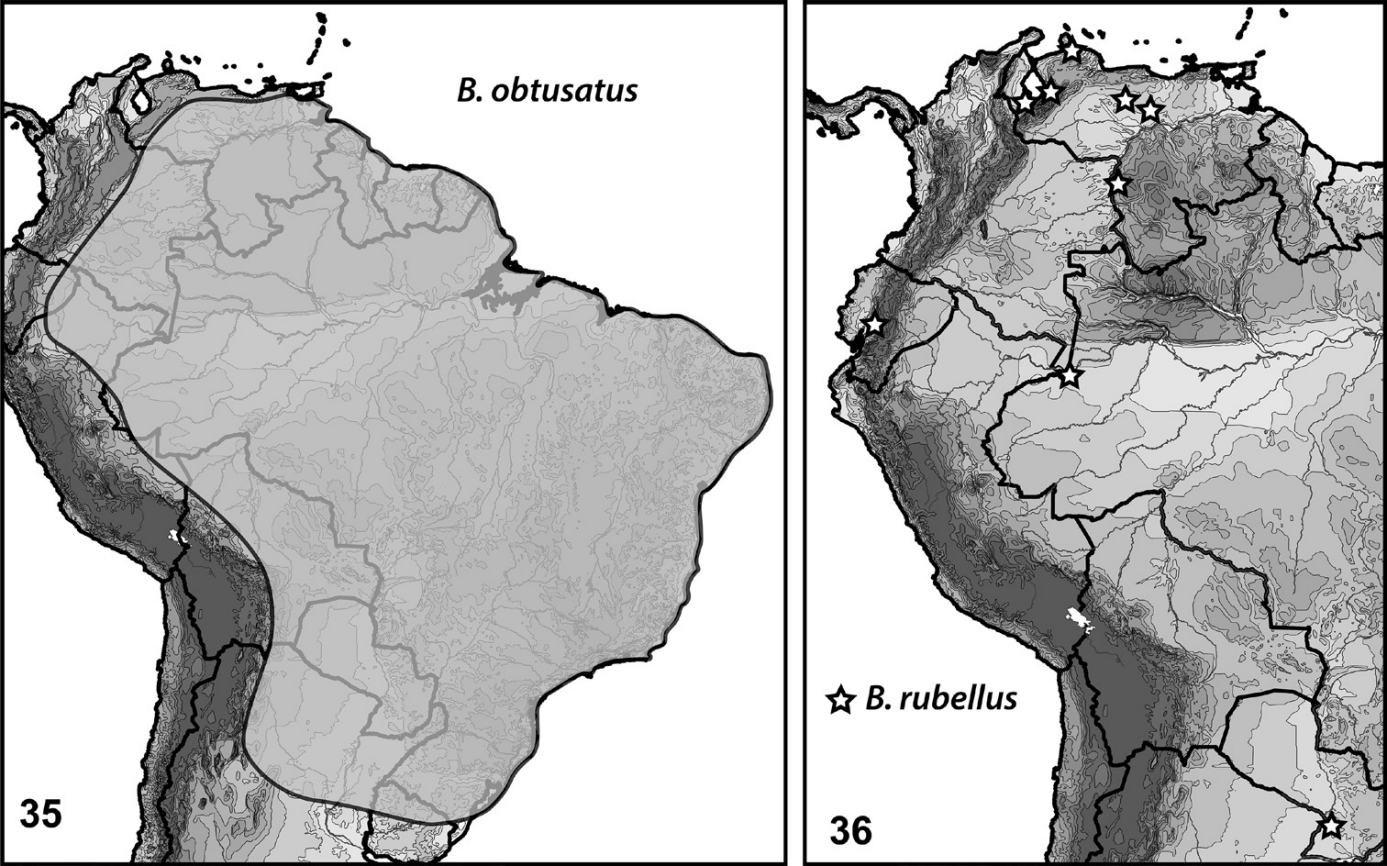
*Bidessonotus* species, South American distributions.

Figs 1 , 2 , 22 , 35

Bidessonotus obtusatus Régimbart, 1895: 336; J. Balfour-Browne 1947: 439; Young 1969: 2; 1990: 363; Biström 1988: 18; Nilsson 2016: 99.
Bidessus (Bidessonotus) obtusatus , Zimmermann 1920: 62.
Bidessus obtusatus , Blackwelder 1944: 76.

#### Diagnosis.

Specimens of this species are rather darkly colored with variable light markings that are usually conspicuous. The anterior clypeal margin is weakly sulcate and medially rounded. The prosternal process is relatively broad, flat and apically narrowly rounded. The male median lobe is diagnostic with the apical blade broadly truncate on the ventral margin, broad dorsally with a prominent obliquely truncate apicodorsal projection and the proximal margin with a small, irregular medial tooth and more well developed dorsal tooth (Fig. 22a,d). The right lateral lobe has the apical segment as long as the basal segment and is elongate and parallel sided and apically narrowly rounded (Fig. 22b). The left lateral lobe is much broader than the right, apically broadly concave with a distinct lobe along the ventral margin (Fig. 22c).

#### Discussion.

This is one of the commonest species in much of lowland South America with specimens collected especially from marshy lentic habitats and at lights. Young (1990) thought the species is related to *Bidessonotus pollostus*, *Bidessonotus rubellus*, *Bidessonotus dubius* and *Bidessonotus ploterus*.

#### Distribution.

Specimens have been collected from throughout lowland South America (Fig. 35). Young (1990) reported seeing hundreds of specimens from Argentina, Brazil, Bolivia, French Guiana, Colombia, Ecuador, Paraguay, Peru, Suriname, and Venezuela. The species is seemingly not as common as others in northern South America, but is present in lowland areas of Venezuela east to French Guiana.

### Bidessonotus otrerus

Taxon classificationAnimaliaColeopteraDytiscidae

Young, 1990

Figs 23 , 32

Bidessonotus otrerus Young, 1990: 368; Nilsson 2016: 99.

#### Diagnosis.

*Bidessonotus otrerus* is a brownish species with significant portions of the head and pronotum brown. The elytra are brown with weakly pale maculae. The prosternal process is narrow and slightly sulcate. The apical blade of the male median lobe is slender and dorsally narrowly rounded, the distal and proximal margins are irregularly undulate (Fig. 23a). The apex of the male median lobe is more obliquely contorted and twisted than it is in other species (Fig. 23a). The basal segment of the right lateral lobe is very large and broad and the apical segment is elongate and slender with the apex rounded (Fig. 23b). The left lateral lobe has the apical segment shorter than the right with the apex broadly and distinctly bilobed (Fig. 23c).

#### Discussion.

Little is known of this species.

#### Distribution.

Young (1990) reported the species from Colombia and Venezuela (Fig. 32). No new additional specimens were examined for this project.

### Bidessonotus paludicolus

Taxon classificationAnimaliaColeopteraDytiscidae

Young, 1990

Figs 24 , 30

Bidessonotus paludicolus Young, 1990: 372; Nilsson 2016: 99.

#### Diagnosis.

Specimens are relatively darkly colored with the posterior surface of the head brown and the elytra evenly dark brown. The prosternal process is narrow and slightly sulcate. The male median lobe has the apical blade narrow ventrally and evenly and broadly expanded dorsally with the dorsal margin broadly truncate (Fig. 24a,d). The right lateral lobe has the apical segment very broad, relatively short and apically broadly rounded (Fig. 24b). The left lateral lobe has the apical segment short, broad and apically very broadly rounded (Fig. 24c).

#### Discussion.

Young (1990) regarded this species as similar to *Bidessonotus mexicanus* from Mexico and Central America, but it is not well known.

#### Distribution.

Young (1990) reported the species from Costa Rica and Mexico, but also from Venezuela (Guarico) (Fig. 30). No additional specimens were examined for this project.

### Bidessonotus ploterus

Taxon classificationAnimaliaColeopteraDytiscidae

Young, 1990

Figs 25 , 31

Bidessonotus ploterus Young, 1990; Nilsson 2016: 99.

#### Diagnosis.

Specimens are relatively evenly brownish on the elytra with poorly-developed maculae. The prosternal process is lanceolate and slightly sulcate. This is a relatively small species (1.3-1.7 mm). The apical blade of the male median lobe is narrow with a relatively well developed anteroventral rounded prominence and the dorsal margin of the blade broadly truncate with a moderately distinctive medial tooth on the proximal margin (Fig. 25a,d). The right lateral lobe has the apical segment elongate, as long as the basal segment, with a prominent lobe on the ventral surface (Fig. 25b). The apical segment of the left lateral lobe is shorter and broader, apically obliquely truncate and apically expanded (Fig. 25c).

#### Discussion.

Young (1990) regarded this species as similar to *Bidessonotus obtusatus*.

#### Distribution.

This species is known from Brazil (Mato Grosso) (Fig. 31, Young 1990). No additional specimens were examined for this project.

### Bidessonotus rubellus

Taxon classificationAnimaliaColeopteraDytiscidae

Young, 1990

Figs 4 , 26 , 36

Bidessonotus rubellus Young, 1990: 366; Nilsson 2016: 99.

#### Diagnosis.

Specimens are relatively darkly colored with the posterior surface of the head brown and the elytra dark reddish-brown with indistinct maculae. The prosternal process is moderately broad, apically rounded and not sulcate. The apical blade of the male median lobe is similar to that of *Bidessonotus obtusatus* but is narrower, has the apicoventral region obiquely truncate and larger with the apicoproximal tooth more strongly developed (Fig. 26a). The right lateral lobe has the apical segment as long as the basal segment and is slender and only slightly expanded apically before narrowly rounded apex (Fig. 26b). The left lateral lobe has the apical segment broad and apically obliquely distinctly bilobed (Fig. 26c).

#### Discussion.

*Bidessonotus rubellus* was thought to have genitalia similar to *Bidessonotus obtusatus* by Young (1990), but inconclusively so. Specimens have been largely collected from lentic habitats and lights at night.

#### Distribution.

*Bidessonotus rubellus* has been reported from Colombia, Ecuador, Panama, Paraguay and Venezuela (Fig. 36, Young 1990). Examined specimens include the following: **Venezuela**: Amazonas, Communidad Porvenir, just S of, 5.341°N, 67.755°W, 15 Jan 2009, Short & Garcia (1, SEMC); Falcon, Medanos de Coro, 11.436°N, 69.668°W, 09 Jul 2009, Short & Shepard (55, SEMC); Guarico, Las Mercedes, approx 65 km S, 8.528°N, 66.376°W, 09 Jan 2009, Short, Garcia, Camacho, Miller (6, SEMC); Trujillo, La Cieba, approx 10 km E, by cemetery, 9.475°N, 70.955°W, 28 Jan 2012, Short, Arias, Gustafson (34, SEMC); Zulia, Encontrados, approx 3 km SE, 9.033°N, 72.212°W, 27 Jan 2012, Short, Arias, Gustafson (16, SEMC).

### Bidessonotus tibialis

Taxon classificationAnimaliaColeopteraDytiscidae

Régimbart, 1895

Figs 3 , 27 , 37

Bidessonotus tibialis Régimbart, 1895: 337; J. Balfour-Browne 1947: 447; Young 1969: 2; 1990: 357; Biström 1988: 18; Nilsson 2016: 99.
Bidessus (Bidessonotus) tibialis , Zimmermann 1920: 62.
Bidessus tibialis , Blackwelder 1944: 76.
Bidessonotus sobrinus J. Balfour-Browne, 1947: 445; Young 1969: 2; 1990: 358; Biström 1988: 18; Nilsson 2016: 99; **syn. n.**

#### Diagnosis.

Specimens have the elytra relatively uniformly-colored except in some specimens with darker and lighter markings, but poorly evident. The prosternal process is lanceolate and distinctly sulcate. The male genitalia are diagnostic with the apical blade moderately slender and distinctly and abruptly curved with the dorsal margin rounded and with a distinct anteroventral rounded prominence (Fig. 27a,d). The right lateral lobe has the apical segment very slender, as long as the basal segment and somewhat expanded apically (Fig. 27b). The right lateral lobe has the apical segment broader, apically obliquely truncate and somewhat expanded (Fig. 27c).

#### Discussion.

This species and *Bidessonotus sobrinus* are extremely similar, and Young (1990) thought they are probably the same species, a conclusion with which I agree. He did not formally synonymize the two, but they are regarded here as subjective synonyms (*Bidessonotus tibialis* Régimbart, 1895 = *Bidessonotus sobrinus* J. Balfour-Browne, 1947, syn. n.). Specimens have been collected mainly from a variety of habitats, both lentic and slow lotic.

#### Distribution.

**Figure 37.**
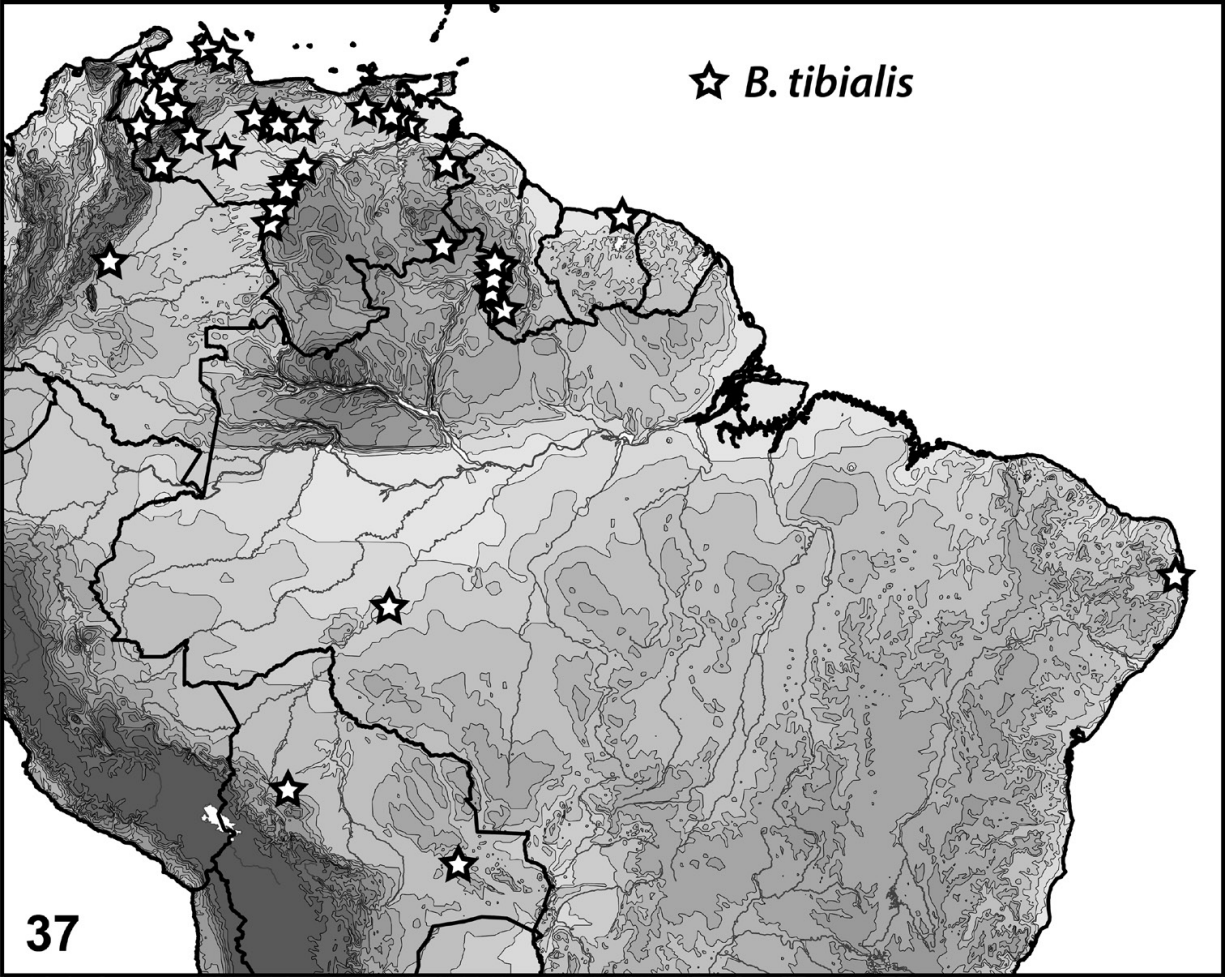
*Bidessonotus* species, South American distributions.

*Bidessonotus tibialis* is widespread in northern South America with records also from Bolivia, Brazil and Peru (Fig. 37) suggesting the species may be more widespread in the continent that current records indicate. Young (1990) reported this species from Brazil, Bolivia and Peru, and (as *Bidessonotus sobrinus*) from Colombia, Panama, Suriname and Venezuela. Examined specimens include the following: **Bolivia**: Dpt Sta Cruz, Prov Chiquitos, mud puddle in road, 1.8km SSW San Jose 17°40'51"S 60°44'33"W, 325m 27 Jun 1999, KB Miller (42, KBMC); Dpt St Cruz, Prov Chiquitos, 2.7km S San Jose, pool in stream, 17°52'20"S 60°44'26"W, 333m, 27 Jun 1999, KB Miller (9, KBMC); Dpt Sta Cruz, Prov Ichilo, 1.2km SSE Buena Vista, marsh, 19 Jun 1999, KB Miller (1, KBMC); Beni; 1.8k E San Borja, muddy pool, 14°52'02"W 66°43'45"W, 15 Jul 1998, KB Miller (1, KBMC). **Brazil**: Cuyaba, Aug (1, KBMC). **Colombia**: Meta, Villavicencio, 10 km S, 4.080°N, 73.684°W, 03 Mar 1969, PJ Spangler (8, USNM). **Guyana**: Pirara Ranch, Caskew Lake, 3.616°N, 59.666°W, 27 Apr 1995, Spangler & Perry (1, SEMC). Region 9, Along road to Parabara, creek crossing at Mushai Wao, 2.159°N, 59.292°W, 01 Nov 2013, Short, Isaacs, Salisbury (2, SEMC); Region 9, Farm pond on ranch, nr. Kusad Mts., 2.853°N, 59.922°W, 28 Oct 2013, AEZ Short (4, SEMC); Region 9, Karanambo, 3.749°N, 59.299°W, 02 Apr 1994, PJ Spangler (12, USNM); Region 9, Katu Wao River near ranch house, nr. Kusad Mts., 2.890°N, 59.850°W, 26 Oct 2013, Short, Isaacs, Salisbury (2, SEMC); Region 9, nr. Kusad Mts., large marshy area, 2.870°N, 59.916°W, 27 Oct 2013, Short, Isaacs, Salisbury (3, SEMC); Region 9, Pooled up creek, tributary of Katu Wao River, nr. Kusad Mts., 2.809°N, 59.865°W, 26 Oct 2013, Short, Isaacs, Salisbury (4, SEMC); Region 9, Ziida Karisihizi (Lake), nr. Kusad Mts., 2.829°N, 59.806°W, 25 Oct 2013, Short, Isaacs, Salisbury (1, SEMC); Region 9, Ziida Wao (Creek), nr. Kusad Mts., 2.828°N, 59.809°W, 25 Oct 2013, Short, Isaacs, Salisbury (32, SEMC); Pirara Ranch, Pirara River, 3.535°N, 59.675°W, 24 Apr 1995, Spangler & Perry (3, USNM). **Suriname**: Para, Paramaribo, 25 km S, 5.578°N, 55.192°W, 12 Jul 1969, PJ Spangler (5, USNM). **Venezuela**: Guarico, Calabozo, 32 km SW, 8.664°N, 67.552°W, 11 Feb 1969, PJ Spangler (4, USNM); Guarico, Hato Masaguaral, 8.566°N, 67.583°W, 06 Mar 1986, Spangler & Beaujon (1442, USNM). Amazonas, Communidad Porvenir, just S of, 5.341°N, 67.755°W, 15 jan 2009, Short & Miller (17, SEMC); Amazonas, Puerto Ayacucho, N, nr Iboruwa, “Tobogancito”, 5.806°N, 67.438°W, 13 Jan 2009, KB Miller (3, SEMC); Anzoategui, Transect 1, 9.293°N, 64.223°W, 15 Aug 2009, R Cordero (1, SEMC); Apure, Mantecal, approx 10 km W, side road, 7.621°N, 69.061°W, 18 Jan 2009, Short, Camacho, Miller (15, SEMC); Barinas, Ciudad Bolivia, approx 13 km SE, large Hacienda, 8.323°N, 70.470°W, 25 Jan 2012, Short, Arias, Gustafson (11, SEMC); Bolivar, between Caicara & Los Pijiguaos, 7.3498°N, 66.298°W, 12 Jan 2009, Short, Camacho, Garcia, Joly, Miller (30, NMPC); Bolivar, Gran Sabana, 1 km E Pauji, tributary of Rio Pauji, 4.479°N, 61.581°W, 16 Jul 2010, Short, Arias, Tellez (4, SEMC); Bolivar, Los Pijiguaos, outcrop/morichal, 6.593°N, 66.820°W, 12 Jan 2009, Short & Miller (6, SEMC); Bolivar, Rio Caripito, 6.586°N, 67.029°W, 12 Jan 2009, Short & Miller (3, SEMC); Bolivar, Tumeremo, E, on road to Bochinche, 7.384°N, 61.325°W, 13 Jul 2010, Short, Arias, Tellez (14, SEMC); Cojedes, El Baul, 5 km S, large marsh, 8.900°N, 68.321°W, 21 Jan 2012, Short, Arias, Gustafson (4, SEMC); Falcon, Medanos de Coro, 11.436°N, 69.668°W, 09 Jul 2009, Short & Shepard (1, SEMC); Falcon, Tocopero, SE of, 11.448°N, 69.218°W, 10 Jul 2009, Short, Camacho, Inciarte, Garcia, Gustafson, Shepard, Sites (6, SEMC); Guarico, Calabozo, 32 km SW, 8.664°N, 67.552°W, 11 Feb 1969, PJ Spangler (12, USNM); Guarico, Las Mercedes, approx 65 km S, 8.528°N, 66.376°W, 09 Jan 2009, Short, Garcia, Camacho, Miller (30, SEMC); Monagas, Chaguaramas, 4 km S, 8.634°N, 62.765°W, 19 Jul 2010, Short, Arias, Tellez (2, SEMC); Monagas, Morichal Largo & Temblador, small pond between, 9.096°N, 62.726°W, 02 Feb 2010, Short, Garcia, Joly (10, SEMC); Monagas, S of Maturin, Morichal at road crossing, 9.273°N, 62.937°W, 02 Feb 2010, Short, Garcia, Joly (5, SEMC); Tachira, La Pedrera, 10 km E, Mata de Limon, small lagoon on finca, 7.502°N, 71.488°W, 26 Jan 2012, Short, Arias, Gustafson (4, SEMC); Trujillo, Granados, approx 3 km SW, 9.376°N, 70.818°W, 28 Jan 2012, Short, Arias, Gustafson (2, SEMC); Zulia, Puente del Zulia, lagoon on finca, 8.551°N, 72.336°W, 27 Jan 2012, Short, Arias, Gustafson (4, SEMC); Zulia, Quebrada Riencito, 10.860°N, 72.322°W, 30 Dec 2008, Short & Garcia (3, SEMC); Zulia, Sabana de Machango, 10.043°N, 71.007°W, 29 Jan 2012, Short, Arias, Gustafson (3, SEMC).

### Bidessonotus truncatus

Taxon classificationAnimaliaColeopteraDytiscidae

J. Balfour-Browne, 1947

Figs 28 , 32

Bidessonotus truncatus J. Balfour-Browne, 1947: 440; Young 1969: 2; 1990: 376; Biström 1988: 18; Nilsson 2016: 99.

#### Diagnosis.

Specimens of this species are darkly colored with the elytra nearly evenly dark brown. The prosternal process is relatively broad, flat, apically pointed, and weakly or not sulcate. The apical blade of the male median lobe is slender with the distal margin medially with an elongate spinous “horn” or process (Fig. 28a,d). The right lateral lobe has the apical segment relatively slender, medially more broadly expanded and as long as the basal segment (Fig. 28b). The left lateral lobe has the apical segment shorter, broadly obliquely concave apically and bilobed (Fig. 28c).

#### Discussion.

Little has been reported about this species, but specimens were collected from both lentic and slow lotic habitats.

#### Distribution.

Known from Bolivia, Brazil, Guayana, Paraguay, Peru, Suriname and Trinidad (Fig. 32, Young 1990). Examined specimens include the following: **Guyana**: Mayuruni Potaro, Takutu Mountains, 6.216°N, 59.049°W, 19 Dec 1983, Spangler, Faitoute, Ed W. (4, USNM). **Venezuela**: Amazonas, Communidad Porvenir, just S of, 5.341°N, 67.755°W, 15 Jan 2009, Short & Garcia (33, SEMC); Amazonas, Road between Puerto Ayacucho and Samariapo, 5.341°N, 67.755°W, 06 Jan 2006, Short, Andrew E (4, SEMC); Bolivar, Gran Sabana, between Kavanayen and Rt 10, 5.741°N, 61.515°W, 01 Aug 2008, Short, Andrew E (6, SEMC); Bolivar, Gran Sabana, N Santa Elena, Rio Guara at Rt 10, 4.622°N, 61.094°W, 17 Jul 2010, Short, Arias, Tellez (2, SEMC); Bolivar, Gran Sabana, N Santa Elena, River at Rt 10 crossing, 4.672°N, 61.068°W, 15 Jul 2010, Short, Camacho, Tellez (4, MIZA); Guarico, Hato Masaguaral, 8.566°N, 67.583°W, 06 Mar 1986, Spangler & Beaujon (8, USNM); Monagas, Morichal Largo & Temblador, small pond between, 9.096°N, 62.726°W, 02 Feb 2010, Short, Garcia, Joly (6, SEMC).

### Bidessonotus vicinus

Taxon classificationAnimaliaColeopteraDytiscidae

J. Balfour-Browne, 1947

Figs 29 , 31

Bidessonotus vicinus J. Balfour-Browne, 1947: 428; Young 1969: 2; 1990: 370; Biström 1988: 18; Nilsson 2016: 100.

#### Diagnosis.

This is a relatively dark and weakly maculate species. The prosternal process is lanceolate, flat and apically pointed. The apical blade of the male median lobe is relatively simple, the anteroventral angle is moderately produced as a broad, curved, short process, the distal margin is broadly truncate to slightly undulate, and there are no other prominent spines, denticles or projections (Fig. 29a,d). The apical segment of the right lateral lobe is medially very broad, short and subtriangular (Fig. 29b). The left lateral lobe has the apical segment subquadrate and very broad (Fig. 19c).

#### Discussion.

Although originally described from a female, Young (1990) clarified the identity of this species which is distributed in Central America and Venezuela.

#### Distribution.

Young (1990) reported this species from Honduras, Panama and Venezuela (Fig. 31). No additional specimens were examined for this project.

### Species in the Genus *Bidessonotus* Régimbart

*Bidessonotus annae* **sp. n.** – Venezuela

*Bidessonotus bicolor* Guignot, 1957 – Brazil

*Bidessonotus browneanus* J. Balfour-Browne, 1947 – Cuba, Dominican Republic, Jamaica, Puerto Rico

*Bidessonotus canis* Miller, 1997 – Costa Rica

*Bidessonotus caraibus* (Chevrolat, 1863) – Belize, Cuba

*Bidessonotus championi* J. Balfour-Browne, 1947 – Costa Rica, Guatemala, Honduras, Nicaragua

*Bidessonotus dubius* Young, 1990 – Brazil, French Guiana, Suriname, Venezuela

*Bidessonotus fallax* J. Balfour-Browne, 1947 – Cuba

*Bidessonotus inconspicuus* (LeConte, 1855) – Canada, USA (eastern states)

*Bidessonotus inigmaticus* Young, 1990 – Mexico

*Bidessonotus josiahi* **sp. n.** – Venezuela

*Bidessonotus longovalis* (Blatchley, 1919) – USA (Alabama, Florida, Georgia)

*Bidessonotus melanocephalus* Régimbart, 1895 – Brazil, Peru

*Bidessonotus mexicanus* Régimbart, 1895 – Belize, Mexico, USA (Texas)

*Bidessonotus mobilis* J. Balfour-Browne, 1947 – Belize, Guatemala, Mexico

*Bidessonotus morosus* J. Balfour-Browne, 1947 – Mexico

*Bidessonotus nepotinus* J. Balfour-Browne, 1947 – Trinidad

*Bidessonotus obtusatus* Régimbart, 1895 – Argentina, Bolivia, Brazil, Colombia, Ecuador, French Guiana, Paraguay, Peru, Suriname, Venezuela

*Bidessonotus otrerus* Young, 1990 – Colombia, Venezuela

*Bidessonotus palecephalus* **sp. n.** – Venezuela

*Bidessonotus paludicolus* Young, 1990 – Costa Rica, Mexico, Venezuela

*Bidessonotus peregrinus* J. Balfour-Browne, 1947 – Panama

*Bidessonotus pictus* Young, 1990 – Costa Rica

*Bidessonotus ploterus* Young, 1990 – Brazil

*Bidessonotus pollostus* Young, 1990 – Belize

*Bidessonotus pulicarius* (Aubé, 1838) – USA (Alabama, Florida, Georgia, Louisiana, Mississippi)

*Bidessonotus reductus* **sp. n.** – Venezuela

*Bidessonotus regimbarti* J. Balfour-Browne, 1947 – Mexico?

*Bidessonotus rhampherens* Young, 1990 – Mexico

*Bidessonotus rubellus* Young, 1990 – Colombia, Ecuador, Panama, Paraguay, Venezuela

*Bidessonotus septimus* **sp. n.** – Venezuela

*Bidessonotus spinosus* **sp. n.** – Venezuela

*Bidessonotus tibialis* Régimbart, 1895 – Bolivia, Brazil, Peru (also Colombia, Panama, Suriname, Venezuela as *Bidessonotus sobrinus*)

= *Bidessonotus sobrinus* J. Balfour-Browne, 1947, **syn. n.**

*Bidessonotus truncatus* J. Balfour-Browne, 1947 – Bolivia, Brazil, Guyana, Paraguay, Peru, Suriname, Trinidad

*Bidessonotus valdezi* **sp. n.** – Guiana, Suriname

*Bidessonotus vicinus* J. Balfour-Browne, 1947 – Honduras, Panama, Venezuela

## Supplementary Material

XML Treatment for Bidessonotus annae

XML Treatment for Bidessonotus josiahi

XML Treatment for Bidessonotus palecephalus

XML Treatment for Bidessonotus reductus

XML Treatment for Bidessonotus septimus

XML Treatment for Bidessonotus spinosus

XML Treatment for Bidessonotus valdezi

XML Treatment for Bidessonotus bicolor

XML Treatment for Bidessonotus dubius

XML Treatment for Bidessonotus melanocephalus

XML Treatment for Bidessonotus nepotinus

XML Treatment for Bidessonotus obtusatus

XML Treatment for Bidessonotus otrerus

XML Treatment for Bidessonotus paludicolus

XML Treatment for Bidessonotus ploterus

XML Treatment for Bidessonotus rubellus

XML Treatment for Bidessonotus tibialis

XML Treatment for Bidessonotus truncatus

XML Treatment for Bidessonotus vicinus
